# Neighbours of cancer-related proteins have key influence on pathogenesis and could increase the drug target space for anticancer therapies

**DOI:** 10.1038/s41540-017-0003-6

**Published:** 2017-01-24

**Authors:** Dezső Módos, Krishna C. Bulusu, Dávid Fazekas, János Kubisch, Johanne Brooks, István Marczell, Péter M. Szabó, Tibor Vellai, Péter Csermely, Katalin Lenti, Andreas Bender, Tamás Korcsmáros

**Affiliations:** 1 Department of Morphology and Physiology, Department of Health Science, Semmelweis University, Budapest, Hungary; 2 Department of Genetics, Eötvös Loránd University, Budapest, Hungary; 3 Earlham Institute, Norwich Research Park, Norwich, UK; 4 Gut Health and Food Safety Programme, Institute of Food Research, Norwich Research Park, Norwich, UK; 5 Centre for Molecular Informatics, University of Cambridge, Cambridge, UK; 6 Department of Medicine and Health, University of East Anglia, Norwich, UK; 7 Department of Gastroenterology, Norfolk and Norwich University Hospitals, Norwich, UK; 8 2nd Department of Internal Medicine, Semmelweis University, Budapest, Hungary; 9 Biometric Research Branch, Division of Cancer Treatment and Diagnosis, National Cancer Institute, National Institutes of Health, Bethesda, MD USA; 10 Department of Medical Chemistry, Semmelweis University, Budapest, Hungary

## Abstract

Even targeted chemotherapies against solid cancers show a moderate success increasing the need to novel targeting strategies. To address this problem, we designed a systems-level approach investigating the neighbourhood of mutated or differentially expressed cancer-related proteins in four major solid cancers (colon, breast, liver and lung). Using signalling and protein–protein interaction network resources integrated with mutational and expression datasets, we analysed the properties of the direct and indirect interactors (first and second neighbours) of cancer-related proteins, not found previously related to the given cancer type. We found that first neighbours have at least as high degree, betweenness centrality and clustering coefficient as cancer-related proteins themselves, indicating a previously unknown central network position. We identified a complementary strategy for mutated and differentially expressed proteins, where the affect of differentially expressed proteins having smaller network centrality is compensated with high centrality first neighbours. These first neighbours can be considered as key, so far hidden, components in cancer rewiring, with similar importance as mutated proteins. These observations strikingly suggest targeting first neighbours as a novel strategy for disrupting cancer-specific networks. Remarkably, our survey revealed 223 marketed drugs already targeting first neighbour proteins but applied mostly outside oncology, providing a potential list for drug repurposing against solid cancers. For the very central first neighbours, whose direct targeting would cause several side effects, we suggest a cancer-mimicking strategy by targeting their interactors (second neighbours of cancer-related proteins, having a central protein affecting position, similarly to the cancer-related proteins). Hence, we propose to include first neighbours to network medicine based approaches for (but not limited to) anticancer therapies.

## Cancer: Key proteins hiding in the neighbourhood

Cancer is considered a systems disease in which the interactors of cancer-related proteins have a key role, also as targets to fight cancer. New therapeutic approaches are needed to improve success rates and to identify suitable proteins as novel, alternative drug targets. We designed a computational approach, combining mutation and differential expression data with network information, to analyse the interactions of cancer-related proteins in colon, breast, liver and lung cancer. We found that first (direct) neighbours, not linked previously to the given cancer type, are similarly important as mutated proteins known to be involved in cancer development. We found 223 drugs already in the clinic targeting these proteins but not yet used against cancer as their oncology relevance was hidden so far. Our observations open up new strategies for target selection and anti-cancer drug discovery.

## Introduction

Cancer is increasingly being considered as a “systems” disease, based on the observation that genetic changes and environmental influence rewire cellular networks during carcinogenesis.^1^ Combinational classical chemotherapies, have been successfully applied against fast proliferating haematological cancers, such as acute myeloid or lymphoid leukaemia.^2^ However, chemotherapy has only shown moderate effect against solid cancers like colon cancer or non-small cell lung cancer.^2^ Hence, even today the most effective therapeutic solution against solid cancers is in many cases of surgery. Although the newest, targeted therapies of solid cancer enhance patient survival, malignant cells often display fast evolution, and thereby develop drug resistance.^3^ Therefore, to achieve a higher success rate in curing solid cancers, new therapeutic approaches are required, such as the identification of suitable proteins that can serve as novel, alternative drug targets for treatment. In the following two paragraphs, we describe two sets of proteins that are in the major focus of current anticancer research: proteins encoded by mutated genes, and proteins having a differential expression in normal and disease states.

The number of mutated genes, which are directly involved in carcinogenesis, is very low compared to those encoded by the whole genome. Vogelstein and his colleagues described 138 so called driver genes,^4^ which are directly involved in cancer progression. The Cancer Gene Census (CGC) database contains 547 such gene across various cancer types.^5^ Remarkably, few driver genes having specific point mutations appear to be sufficient to rewire signalling networks in cancer,^1^ which at the same time shows that—at least from the mutational side—cancer does not consist of an “infinite” number of different diseases, and in many cases treatment options targeted against driver genes might be transferred from one case to the next. Biological knowledge and network-based approaches have been developed to understand the mechanisms of driver gene influence. Pathway analysis^6–8^ showed that most driver genes are part of central signalling pathways, like MAPK, TGF-β, JAK/STAT, Notch, Hedgehog and WNT,^4,9^ which are implicated in carcinogenesis, growth and differentiation. However, in most cases, pathway analysis does not explain why some pathway members are much more often found to be mutated than others. To understand the selection mechanism behind mutations, network-based studies were used to estimate the importance of a mutated protein compared to non-mutated ones in signalling and protein–protein interaction networks.^10–13^ Proteins mutated in cancer were found having a high number of interacting partners (i.e., a high degree of connectivity), which indicates high local importance.^10^ Mutated proteins are also often found in the centre of the network, in key global positions, as quantified by the number of shortest paths passing through them if all proteins are connected with each other (i.e., they have high betweenness centrality; hereafter called betweenness).^11,12^ Mutated proteins also have high clustering coefficients, which means their neighbours are also neighbours of each other.^10,13^ Moreover, neighbourhood analysis of mutated proteins have been previously successfully used to predict novel cancer-related genes.^14,15^ However, to the best of our knowledge, no study has concentrated particularly on the topological importance of first neighbours of mutated proteins in cancer, and their usefulness as drug targets themselves.

The other frequently studied group of genes in cancer biology is the set of differentially expressed genes (DEGs). Since microarray and next generation sequencing data became widespread, an increasing number of genes were found to differ in expression between cancer systems and healthy cells, either by upregulation or by downregulation.^16,17^ To find the most relevant DEGs for disease occurrence and progression, one approach is to select those DEGs that have the most central position in the network.^18,19^ We note that these studies do not take into account the interaction neighbourhood of DEG coded proteins if the neighbour proteins have unchanged expression. Pathway analysis is another approach to prioritize DEGs by identifying those DEGs that have been annotated to function in cancer-related pathways.^20^ Although here the interactions of a DEG coded protein provide the evidence for the pathway function, network and pathway analysis based studies also do not consider the network parameters or neighbourhood of proteins coded by DEGs. The Cancer Genome Atlas (TCGA) and the International Cancer Genome Consortium studies extended the scope of DEG analysis by joint examination with mutated proteins.^21^ These studies were successful to find different disease clusters in solid cancers.^17,22^ Nonetheless, these studies have not yet focused on the role of first neighbours in cancer, other than that first neighbours could connect cancer-related proteins.^23^

Prompted by the lack of a focused analysis of the neighbourhood of cancer-related proteins, we compared the network features of mutated genes and DEG coded proteins with their first neighbours. We also aim to investigate whether these first neighbour proteins could be considered as a potential set of novel anti-cancer drug targets in particular in the area of solid cancers, which are in dire need of new treatment modalities. To provide generalizable results, we selected four solid cancer types with high mortality rate, namely colon, breast, and hepatocellular carcinoma (HCC), as well as non-small cell lung cancer (NSCLC). Given that the aim of this study was to investigate general trends among first neighbours of cancer-related proteins, all known subtypes of these cancers were included in the analysis, listed in Supplementary Table 1. For each examined cancer type, we then measured the network parameters of cancer-related proteins and their first neighbours in multiple protein–protein interaction and signalling network resources and were able to show first neighbours have as high network centrality parameters as cancer related proteins themselves. This led us to evaluate the therapeutic applicability of the first neighbours in anti-cancer treatment. To select the most relevant first neighbour proteins for oncology, we suggest and provide examples for two complementary strategies: (1) a drug target discovery approach focusing specifically on the first neighbours of differentially expressed proteins and presenting a network medicine-based target selection approach; and (2) a drug repurposing approach based on analysing existing drugs and compounds.

## Results

### Identification of cancer-related proteins and their first neighbours

We considered a protein cancer-related, if it was mutated or had a differential expression in cancer. We collected mutation and expression data from the CGC^5^ and the Gene Expression Omnibus (GEO)^24^ resources, respectively. We defined a protein differentially expressed in a given tissue, if the corresponding mRNA was either present only in normal tissue and absent in cancerous tissue, and vice versa. After calculating the mean and standard deviation of our datasets, we determined proteins with expression levels below the mean minus the standard deviation as not expressed to discretise the gene expression to an on/off value (see Methods for details). By combining the lists of mutated and differentially expressed genes, for each examined cancer type separately, we defined a protein as cancer-related if (1) its coding gene was listed in the CGC as mutated in the given cancer type, and/or (2) its corresponding mRNA was found to be differentially expressed between the control and cancer tissues. We constructed tissue-specific networks from the differential expression data as well to examine the network effect of differently expressed cancer-related proteins. An interaction from a signalling or protein–protein interaction network was valid if both interactors from the given network were expressed in a given tissue, regardless it was normal or cancer.

First neighbours of cancer-related proteins were defined as proteins (1) directly and physically interacting with cancer-related proteins in human interactomes and signalling networks according the network databases used (see below); and (2) which were not cancer-related proteins themselves. We also defined as “unaffected” those proteins that are neither first neighbours of cancer-related proteins, nor cancer-related proteins themselves in the given cancer type. The cancer type specific analysis is important as some proteins classified as unaffected in one cancer type can be cancer-related in another cancer type. Accordingly, it is worth distinguishing from the many unaffected proteins those that have a directed interaction towards a first neighbour as they have a similar position as a cancer-related protein. We termed these distinctive proteins as influencer proteins.

For the interaction and network data we used three detailed signalling network resources, SignaLink 2,^25^ Reactome,^26^ and a cancer signalling network compiled by Cui *et al*.^10^, as well as two more global protein-protein interaction (PPI) networks, namely the manually curated HPRD,^27^ resource and the integrated dataset comprising DIP,^28^ IntAct,^29^ and BioGrid.^30^ All these networks have different compilation protocols and thereby provide partially different information. We performed all analyses separately with each network resource, to avoid the study and curation bias of using a single resource and to thereby provide more general conclusions from this work. We developed cancer-specific and tissue-specific networks by combining expression datasets with network information (see Methods for details). We listed the protein classifications for each cancer type in Supplementary Table 2.

### First neighbours of cancer-related proteins have high local and global centrality in the network

**Fig. 1:**
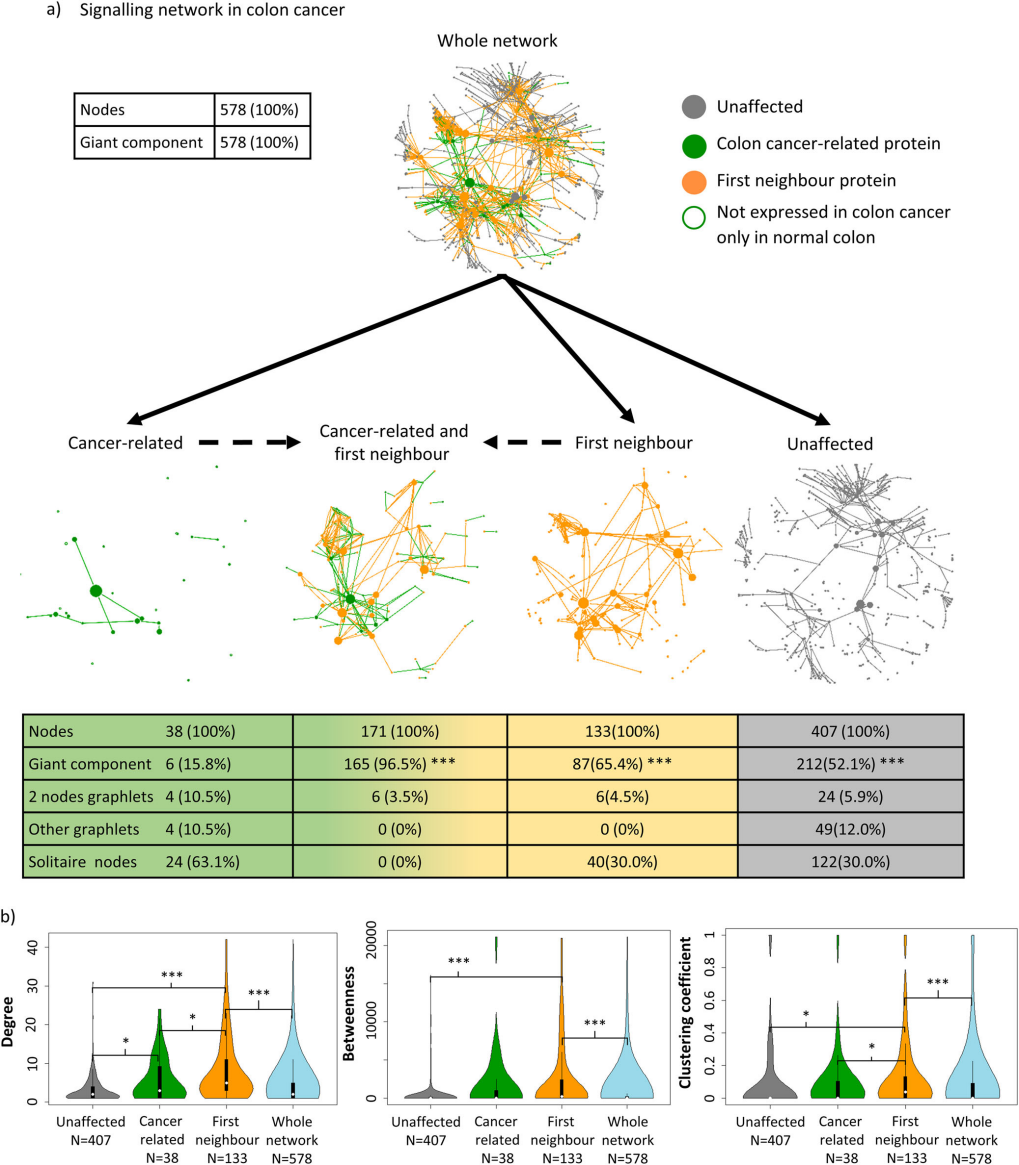
First neighbours have a central position in cellular signalling networks. **a** The signalling network of colon cancer (interactions based on the SignaLink resource). Node sizes are proportional to their betweenness centrality network parameter. Green nodes represent cancer-related proteins, orange nodes are their first neighbours and grey nodes represent the proteins that do not belong to any of these categories (here termed as “unaffected” proteins). Based on cancer-relatedness, sub-graphs have been created, with their major network properties shown below them. Note the different ratio of nodes in the giant component, which is the largest connected graph. (Exact test *** *p* < 0.001) **b** Distribution of network topological properties for each subgraph: degree (local centrality), betweenness centrality (global centrality), and clustering coefficient (neighbourhood connectivity). White dots represent median values, boxes stretch from the 25th percentile to the 75th percentile, and whiskers are twice the length of the boxes. Wilcoxon rank sum tests have been used for statistical testing, with the whole network serving as control. The level of significance is represented using the following scale: *p < 0.05, **p < 0.01, ***p < 0.001

We found cancer-related proteins in locally and globally central positions of networks across each cancer types (measured as degree and betweenness, respectively) in agreement with previous studies.^10–13,18,19^ Throughout the main text, we show the results for colon cancer from the SignaLink network resource. All results for the other three cancer types with the other four network resources are shown in the supplementary materials. Interestingly, most of the cancer-related proteins are not directly interacting with each other, i.e.*,* cancer-related proteins do not form one giant component in the graph (Fig. 1a. and Supplementary Table 3; Exact test *p* > 0.05). Meanwhile, first neighbours of the cancer-related proteins form a larger giant component within the network than expected by chance compared to the same amount of randomly selected proteins in the same network (Fig. 1a, Exact test *p* < 0.001; for the other cancer types and other databases, see Supplementary Table 3; for the methodological details, see Methods). Cancer-related proteins are connected to each other by their first neighbours, and these first neighbours also connect the unaffected proteins. In other words, to signal to another cancer-related protein, and further parts of the signalling network, cancer-related proteins make extensive use of their first neighbours (Fig. 1a). Therefore, the rewiring effect (i.e*.*, the number of affected biological processes) of cancer-related proteins is significantly higher, if we consider those processes that they reach through their first neighbours (*p* < 0.001 Bernoulli test; see Supplementary Table 4 and Methods for details). For example, through their first neighbours, colon cancer-related proteins affect important processes implicated in carcinogenesis, such as angiogenesis, autophagy, and DNA repair that are not direct functions of the cancer-related proteins themselves.

In a similar fashion as cancer-related proteins, their first neighbours also have higher degree, higher betweenness and higher clustering coefficient, compared to either the whole network or to the proteins unaffected by cancer (*p* < 0.001 in both cases, Wilcoxon rank sum test; Fig. 1b, Supplementary Table 5). Remarkably, compared to colon cancer-related proteins, first neighbours have higher degree (*p* < 0.05, Wilcoxon rank sum test), similar betweenness and higher clustering coefficient (*p* < 0.05, Wilcoxon rank sum test). Likewise, we found similar significant differences in the network topology parameters of first neighbours in the other three cancer types (breast, HCC, NSCLC) (Supplementary Fig. 1. and Supplementary Table 5). In addition, examining the other four (signalling and PPI) network sources provided similar results to those from the SignaLink network (Supplementary Table 5, and Supplementary Fig. 2). There was only one minor exception; in the Reactome network the clustering coefficient was not found to be different between the first neighbours and the unaffected proteins (Supplementary Fig. 2b), due to the high number of protein complexes within this database.^26^

We also measured the network centrality parameters in the non-tissue specific (original) networks. We found similar significant differences to those measured in tissue-specific networks, indicating that not only in context specific networks, but in molecular networks in general we can observe the differences of the network parameters of cancer-related proteins and their first neighbours.

Next we tested with a randomization method the robustness of first neighbour selection (see Supplementary Method). Encouragingly, we found that in all four cancer types, the “real” first neighbours were listed as first neighbours in the randomly generated set more than the unaffected proteins (*p* < 0.01, Wilcoxon rank sum test; Supplementary Fig. 3). Similarly, in three out of the four cancer types, the “real” first neighbours were found as first neighbours in the randomly generated set more than the cancer-related proteins (*p* < 0.05; Wilcoxon rank sum test; Supplementary Fig. 3). The only exception was NSCLC, where the reason for the non-significant difference was due to three NSCLC-related proteins (RET, EGFR, PPARγ) having a high degree (i.e*.*, many first neighbours expressed in the lung). In the other cancer types, these three proteins are not cancer-related but first neighbours or unaffected proteins, except for PPARγ, which is cancer-related in breast cancer. Thus, we confirmed that most of the identified first neighbour proteins can be listed based solely on the given network, and their list is independent of the initial set of cancer-related proteins. This observation emphasizes the relevance of integrating expression data with interaction networks to generate cell and context specific networks for such analysis.

### Mutated proteins directly, differentially expressed proteins indirectly, through their first neighbours affect biological networks

**Fig. 2:**
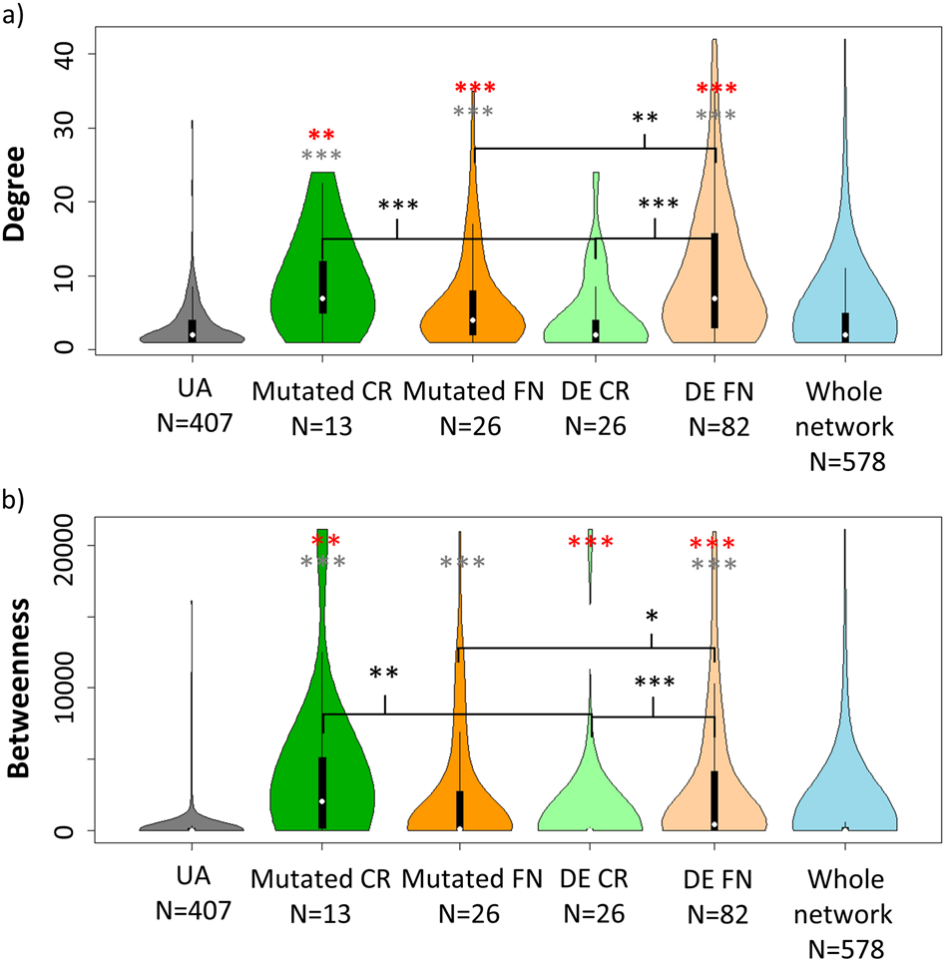
Differences between first neighbours of mutated and differentially expressed proteins in colon cancer. **a**,**b** Violin plot showing the degree and betweenness centrality of mutated or differentially expressed (DE) cancer-related (CR) proteins and their respective first neighbours (FN) to unaffected proteins (UA) or to the whole network in the signalling network of colon cancer (using the SignaLink 2 database). White dots represent the median values. Boxes stretch from the 25th percentile to the 75th percentile, while the whiskers are twice the length of the boxes. Wilcoxon rank sum tests was employed for the statistical analysis, with the unaffected proteins (*grey* stars) and the whole colon cancer specific network (*red* stars) as control. The level of significance is represented using the following scale: *p < 0.05, **p < 0.01, ***p < 0.001. The plots show that FN of DE proteins have higher network centralities than their cancer-related counterparts, meanwhile mutated CR proteins and their FN have similar centrality. *Dark green*—mutated CR proteins, *light green*—DE cancer-related proteins; *orange* nodes indicate the FN of mutated proteins, while *light orange* nodes are the FNs of DE proteins

Encouraged by the finding that first neighbours of cancer-related proteins display a central network position, we investigated the relation between the network topology parameter of a cancer-related protein and its first neighbour proteins. From this analysis we found that cancer-related proteins have two distinct topology patterns both in signalling and PPI networks: Mutated proteins have a higher or same degree, betweenness and clustering coefficient parameters compared to their first neighbours, while differentially expressed proteins have lower degree and betweenness than their first neighbours (Fig. 2a–b. and Supplementary Figs 4, 5; see Supplementary Table 6. for all detailed statistics). Thus, the network position of cancer-related proteins and the topological parameters of their first neighbours are both associated with the alteration type (mutation or differential expression) of the cancer-related protein. We could formalise this observation as two diverse, complementary strategies in carcinogenesis: proteins with high network topology parameters have a high chance to be either mutated themselves, or to be indirectly affected through differentially expressed proteins.

We conclude from using integrative network analysis of four different cancer types in three signalling and two PPI databases that first neighbours (1) have central positions in signalling and PPI networks; (2) connect cancer-related proteins; (3) act like “glue” of the cancerous network to form a giant component; (4) have a potential role in transducing a malignant effect of a cancer-related protein to alter cellular functions; and (5) allow differentially expressed proteins to exert their effects more extensively. Based on these observations, we now suggest a strategy for disrupting cancer-specific interactomes and signalling networks by targeting first neighbour proteins of cancer-related proteins, especially the first neighbours of differentially expressed proteins.

In the following we will provide two complementary ways and examples for selecting the most potent first neighbours of cancer-related proteins as drug targets: (1) A drug target discovery approach focusing on first neighbours of differentially expressed proteins for selecting novel drug targets from scratch; and (2) A drug repurposing approach based on analyzing existing drugs and compounds targeting first neighbours of cancer-related proteins.

### Selecting novel anticancer targets using first neighbours of differentially expressed proteins

While (mutated) proteins with high network topology parameters have been the focus of drug discovery efforts in the past,^31^ the results from the current study suggest first neighbours of differentially expressed proteins play a similarly central role in cancer networks. Hence, we propose first neighbours of differentially expressed proteins as a novel way of selecting anticancer drug targets in the future.

In colon cancer, there are 82 proteins classified as first neighbours of differentially expressed proteins. To evaluate the oncological relevance of these proteins, and also to validate our classification process, we searched for scientific publications regarding colon cancer and these 82 first neighbour proteins. We found and manually checked 1820 publications. The validation part of our analysis can be found in the Supplementary Notes. As for the oncological relevance analysis of these 82 proteins, we found 38 proteins (46%) to have indirect (31 proteins) or, in specific cases, direct (7 proteins) implications in carcinogenesis. Thus, nearly half of the first neighbours of differentially expressed proteins are already been known or suspected to be associated with colon cancer, while the other half (44 proteins) could be considered as novel genes potentially relevant for anticancer drug target discovery.

**Fig. 3:**
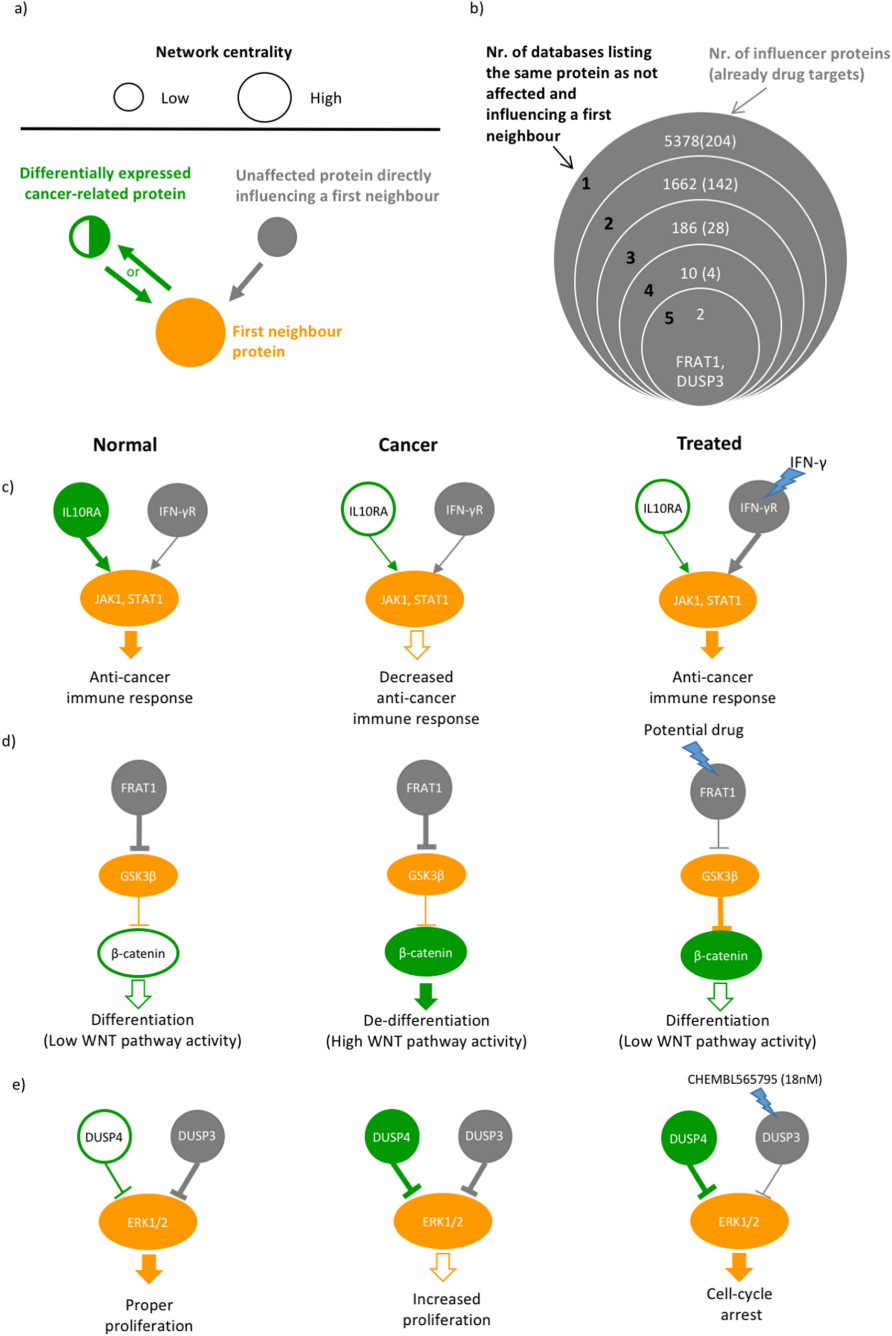
The potential role and number of influencer proteins. **a** The model of action of an influencer protein, which directly affects a first neighbour of a differentially expressed cancer-related protein. Network centrality differences are indicated by the size of the circles. Influencer proteins having lower network centrality parameters could be better drug targets than those first neighbours that are too central and multi-functional. **b** The number of influencer proteins of colon cancer in the overlap of the five network resources used in the current study. The number of proteins that are already drug targets are shown in parenthesis. **c–e** The effect of three influencer proteins, IFN-γR, FRAT1 and DUSP3 in the JAK/STAT, WNT and MAPK signalling, respectively, in normal, cancer and treated cases. At all three examples influencer proteins could be better targets than the first neighbours of differentially expressed cancer-related proteins, which are too central proteins making them difficult to pharmacologically target. Note the colour codes for the proteins: *Green* node: cancer-related, expressed protein; *green* empty node: Cancer-related, not expressed protein; *orange* node: first neighbour protein; *grey* node: unaffected protein, influencer; *arrow*: stimulation; *blunted arrow*: inhibition. For the sake of clarity we present the heterodimer of IFN-γR1 and IFN-γR2 as “IFN-γR”

However, as we pointed out previously, the first neighbours of differentially expressed proteins often have high degree and/or high betweenness parameters, thereby their pharmacological targeting could produce more side effects.^32,33^ Accordingly, these proteins are rarely found in any cancer type mutated or differential expressed, probably because most of them (such as SMAD3, GSK3β, ERK1) are multi-functional and have a central position. Therefore, to pharmacologically target these central first neighbours, we suggest a cancer mimicking, indirect approach: the less central interactors of these first neighbours could serve as reasonable drug targets. In other words, either a differentially expressed protein itself or other interactors of the first neighbour could affect the central first neighbour protein with fewer side effects. This network based reasoning is in agreement with current drug target selection attempts focusing on differentially expressed proteins based only on expression analysis.^34^ Moreover, by using interaction data we can also extend the target space with proteins classified in the first part of the Result section as influencer proteins: non cancer-related or first neighbour proteins that have a directed interaction towards a first neighbour. By this network position, influencer proteins could efficiently affect these first neighbour proteins, and thereby, they could be relevant novel anticancer drug targets (Fig. 3a). Note that by their definition influencer proteins are two steps away from a differentially expressed protein, thus, their identification requires the integration of expression data and interaction data.

In order to investigate current research efforts into the direction proposed here, we investigated the potential list of such influencer proteins in colon cancer. To avoid the interaction bias of one resource and to provide more confident candidates, we combined the five network resources we used in this study. We listed the influencer proteins having at least one directed interaction to a first neighbour of differentially expressed proteins in colon cancer (Supplementary Table 7). Note that with this approach influencer proteins having interaction to different first neighbours in the different resources were also listed emphasizing the relevance of these influencer proteins. We pointed out that influencer proteins in colon cancer found in at least three different resources could provide a reasonable number of proteins (197) for further investigations (Fig. 3b, and Supplementary Fig. 6 for the other cancer types). Supporting the oncological relevance of influencer proteins, we found 128 drugs available against influencer proteins and that is higher than expected by chance (*p* < 0.001 *χ* ^2^ test compared to rest of the proteins in the databases). From the 128 drugs, 62 drugs are already under clinical trial against cancer, and two (Dasatinib and Vandetanib) are currently used in practice according to our literature survey. Interestingly, half of these drugs are different ion channel inhibitors, and most of them could be used in specific cancer types. Such example is metformin, which causes energy deficiency both in colon cancer cell lines and in prostate cancer^35,36^ by targeting different NADH dehydrogenases (influencer proteins) that are interacting with the Cytochrome b-c1 complex (first neighbours of many cancer-related proteins). Interferon gamma (IFN-γ) could also be an interesting treatment option targeting influencer proteins, and as an immunotherapeutic agent, it is under trial in colon cancer with combination of 5-fluoracil or leucovirin.^37^ We present a detailed example with IFN-γ and its receptor, IFN-γR below.

### Examples of the cancer-mimicking strategy

By analyzing the list of all influencer proteins in all the four examined cancer types, we highlight here examples for those influencer proteins that were found in the highest number of network resources. We present three specific cases in colon cancer, and three other examples for each of the other three cancer types we examined. IFN-γR a known drug target we found as an influencer protein in four databases and FRAT1, a protein listed as influencer in all five databases but not yet a drug target demonstrate two different types of connectivity pattern in colon cancer (Fig. 3c–d): (1) the influencer protein (IFN-γR) has the same position as the cancer-related protein; (2) the influencer protein (FRAT1) is affecting the cancer-related protein through the first neighbour.

IFN-γR is a heterodimer of IFN-γR1 and IFN-γR2 that form the receptor of the Interferon-γ cytokine, which is conventionally associated with antitumor mechanisms during cell-mediated adaptive immune responses.^38^ Another key cytokine receptor in these immune responses is the IL10 receptor (IL10R), which is highly expressed in the normal colon, but missing in colon cancer. Both receptors are transducing their effect directly through JAK1 and STAT1 proteins^38^ that we found having central network positions in the examined networks. In cancer, the lack of IL10R decreases the production of antitumorigenic cytokines, and thereby also the activity of the IFN-γ pathway. As IFN-γR is also expressed in cancer and directly connected to the same first neighbours (JAK1 and STAT1) as the cancer-related IL10R, IFN-γR may substitute the role of IL10R upon IFN-γ treatment (Fig. 3c). Accordingly, IFN-γ has been used clinically to treat a variety of malignancies.^38^ IFN-γ treatment is not always beneficial, and some clinical trials against melanoma pointed out that signalling context and tumour microenvironment factors could even turn IFN-γ to a pro-inflammatory and thus a carcinogenic factor.^38^ Nevertheless, studies agree that it could be a viable new therapeutic target for a subset of malignancies.^38^

FRAT1 (Frequently Rearranged in Advanced T-cell lymphoma 1) is a known proto-oncogene in some cancer types that promotes the WNT signalling pathway by inhibiting GSK3β-mediated phosphorylation of β-catenin.^39,40^ In normal colon, the expression level of β-catenin is low, but its expression in cancer is high, causing dedifferentiation. GSK3β, the first neighbour of β-catenin is a central, highly multi-functional protein known as a key protein difficult to inhibit without causing side effects and toxicity.^41^ In glioblastoma and NSCLC cancer, previous studies found that the decreased level of FRAT1 influences the GSK3β activity to phosphorylate β-catenin and by that, inhibit the WNT pathway.^39,40^ The role of FRAT1 in colon cancer is less known but based on its function in other cancer types and its special influencing position in colon cancer signalling, we point out its relevance as a potential target in colon cancer therapy (Fig. 3d). FRAT1 was one of the two proteins that have this special position in all the five examined network sources (Fig. 3b). Nonetheless, a recent in vitro study showed a somehow opposite role for FRAT1 in a metastasis suppressing pathway that highlight the importance of stage and context specific treatments as well as the need for detailed and in vivo studies.^42^

DUSP3 (DUal Specificity protein Phosphatase 3) was the other influencer protein that we found in all five databases for colon cancer. DUSP3 is an influencer protein because it acts on ERK1 and ERK2,^43^ two first neighbours of a colon cancer-related protein, DUSP4, which is only expressed in colorectal cancer cells and not in normal colon cells (Fig. 3e). DUSP3 and DUSP4 are both dual specific phosphatases that dephosphorylate tyrosine and threonine residues and inhibit ERK1 and ERK2. ERK1 and ERK2 are both high network centrality first neighbours and they transduce cell proliferative as well as pro-, and anti-apoptotic signals in a coordinated manner in the MAPK pathway.^44^ The cancer-related DUSP4 overexpression blocks ERK1/2 signalling, and this leads to increased cell proliferation in colorectal cells.^45^ Expression of DUSP3 is needed during intensive cell proliferation to inhibit the active MAPK signal, and blocking DUSP3 by compounds leads to cell cycle arrest.^46,47^ Therefore, DUSP3 could be a potential drug target in colon cancer, similarly as it was proven in cervical cancer, where targeting DUSP3 by small molecules led to decreased proliferation.^48^ The concentration of compounds targeting DUSP3 was in nanomolar IC50 range (Fig. 3e), according to ChEMBL, which makes them promising leads.^48^

**Fig. 4:**
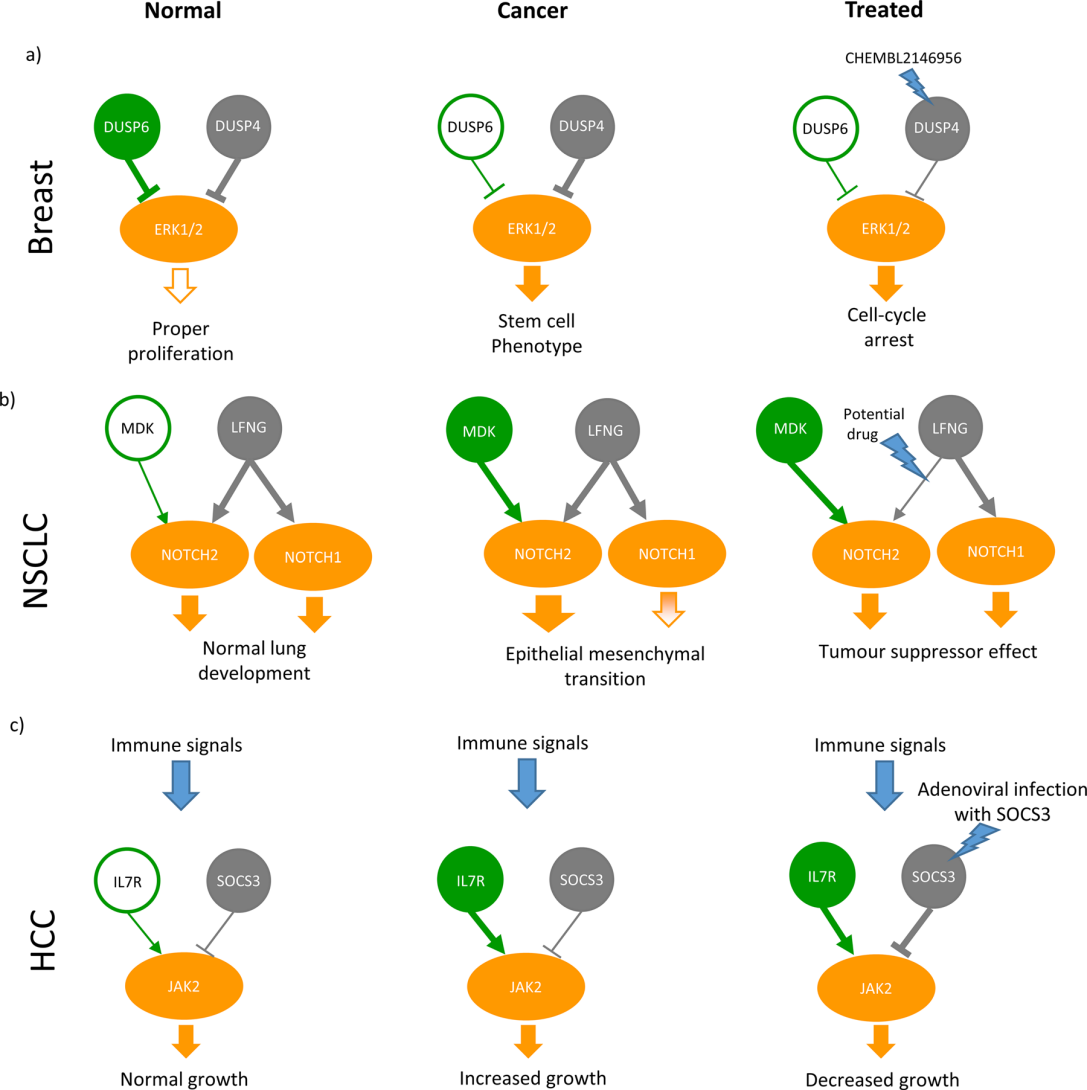
Influencer proteins in breast cancer, NSCLC and HCC. **a–c** Effect of three further influencer proteins, DUSP4, LFNG and SOCS3 in breast cancer, non-small cell lung and in hepatocellular carcinoma, respectively. As in Fig. 3, we compared the probable effect of the influencer proteins on cancer-related proteins in normal, cancer and treated cases. DUSP4 effects ERK signalling (**a**), LFNG stimulates the Notch pathway (**b**), while SOCS3 is an inhibitor of the JAK/STAT pathway (**c**). Note the colour codes for the proteins: *Green* node: cancer-related, expressed protein; *green* empty node: Cancer-related, not expressed protein; *orange* node: first neighbour protein; *grey* node: unaffected protein, influencer; *arrow*: stimulation; *blunted arrow*: inhibition

In breast cancer, DUSP4 is not a cancer-related protein but it is situated in an influencer position according to all five databases. In breast, DUSP4 acts also on ERK1/2 along with a breast cancer-related protein, DUSP6 (Fig. 4a, Supplementary Fig. 6). During the progression of breast cancer, DUSP6 expression decreases according to our and others data.^49^ Thus, during cancer progression due to the less active DUSP6, ERK1/2 becomes more active. Interestingly, DUSP4 alone can still limit the ERK1/2 over activation leading to cancer stem cell formation and epithelial mesenchymal transition (EMT) instead of cell-cycle arrest.^50^ The inhibition of DUSP4 could prevent the EMT in breast cancer,^50^ as well as causing cell cycle arrest, similarly as the inhibition of DUSP3 in colorectal cancer.^46^ Therefore, DUSP4 inhibitors (such as the CHEMBL 2146956 compound, which inhibits DUSP4 with an IC50 of 2.29 μm) are under intensive experimental investigations.

In NSCLC, a Notch pathway modulator called LFNG (lunatic fringe) was found as an influencer in all the five databases (Fig. 4b, Supplementary Fig. 6). LFNG can effect the incoming signals of both NOTCH1 and NOTCH2 receptors in a context dependent manner.^51^ NOTCH2 is a first neighbour of the NSCLC-related Midkine (MDK) protein, which is expressed only in NSCLC and not in normal lung, and it is a known NSCLC biomarker.^52^ MDK can also cause neuroblastoma through activating specifically NOTCH2.^53^ LFNG acts like a double-edged sword: by promoting both the Delta1 ligand activated NOTCH1/2 receptors and decreasing the Jagged1 ligand activated NOTCH1 signalling, it contributes to the normal and tumour suppressor effect of NOTCH1.^51^ However, if the NOTCH2 specific MDK is expressed (as in NSCLC) and acts together with LFNG, the overall role of LFNG is shifted to promote cancer progression, mostly through a NOTCH2 dependent EMT.^54^ A drug selectively targeting the NOTCH2 activating function of LFNG may alter the malignant effect of MDK. The application of such “edgetic” drugs has increased in recent years, especially for difficult but promising drug targets (like LFNG).^31,55^ This edgetic drug has the potential to promote and maintain the tumour suppressive inhibition of LFNG on the Jagged1-NOTCH1 signalling, while blocking the LFNG-NOTCH2 stimulatory interaction. This NOTCH1 dependent tumour suppressive effect of LFNG was confirmed in pancreatic cancer supporting that LFNG could be a potential target of specific anti-cancer treatments.^56^

In HCC, we found SOCS3 (Suppressor Of Cytokine Signalling 3) as an influencer of the JAK2 signalling in all five databases (Fig. 4c, Supplementary Fig. 6). JAK2 is a first neighbour with high network centrality parameters of the HCC-related IL7R protein. IL7R is expressed in HCC but not in normal liver cells, and can activate JAK2 to increase cell growth.^57^ SOCS3 is a negative regulator of the JAK/STAT signalling pathway, and can inhibit JAK2 itself.^58^ Therefore, inhibition of JAK2 by SOCS3 could result in decreased proliferation. A possible way to increase SOCS3 expression in HCC would be using adenoviral infection of hepatocellular carcinoma cells containing the SOCS3 gene. This was found to cause lysis in hepatocellular carcinoma cell lines, but not in normal liver cells indicating that further experimental studies on SOCS3 could increase its future applicability as a cancer cell specific anti-cancer agent.^59^

### Drugs already targeting first neighbours of cancer-related proteins: a drug repurposing approach

**Fig. 5:**
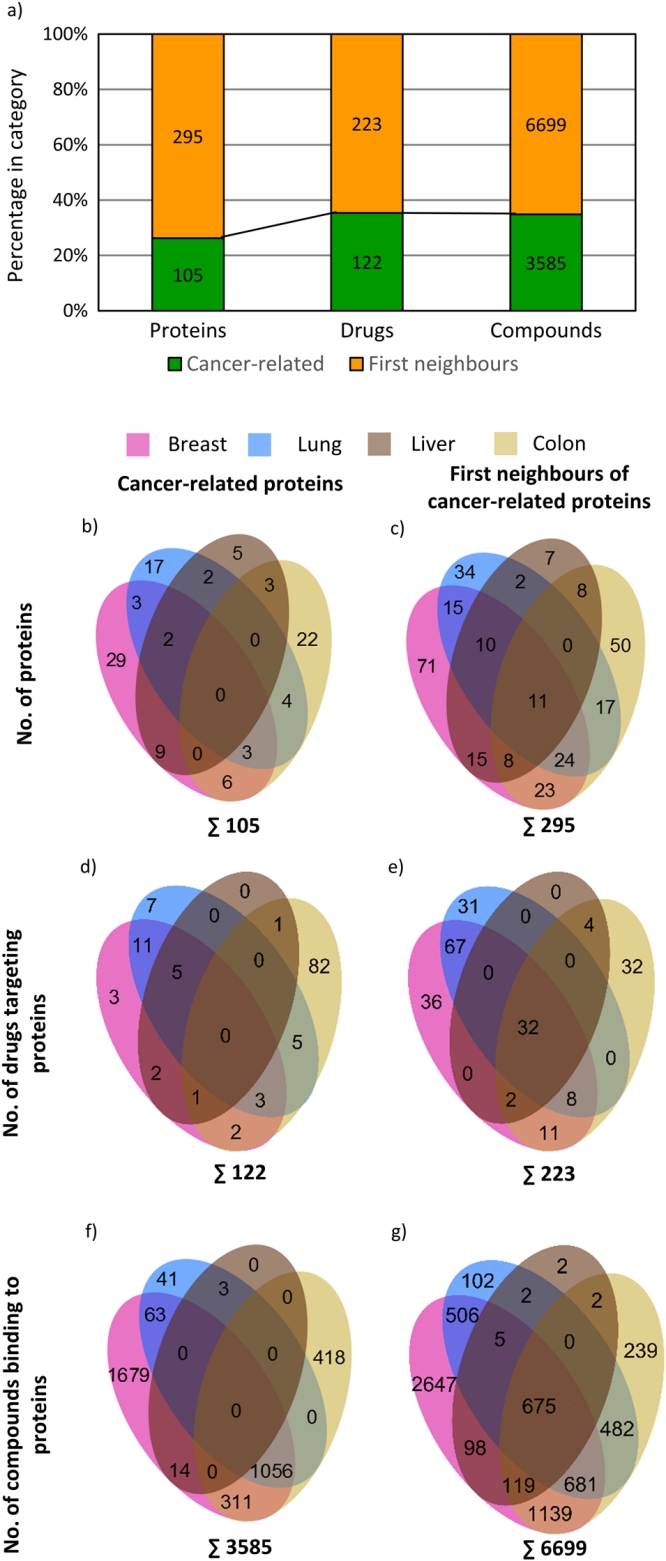
Number of proteins involved in a particular cancer, as well as drugs and compounds acting on cancer-related and their first neighbour proteins. **a**, Stacked columns show the number of proteins involved in a particular cancer, as well as the number of drugs and compounds acting on cancer-related proteins and their first neighbours, respectively. All stacked columns were compared to the cancer-related/all proteins ratio (Bernoulli test: *p* < 0.001). Focusing on first neighbours could provide a twofold increase of testable drugs and compounds for anticancer therapy. **b–g** The venn diagrams show the distribution of available drugs and compounds against the different cancer types. The colours represent each examined cancer type: *pink* standing for breast, *blue* for NSCLC, *brown* for HCC and *tan* for colon cancer. **b** The distribution of cancer-related proteins. **c**, The distribution of first neighbour proteins. **d,** The distribution of drugs against cancer-related proteins. **e**, The distribution of drugs against first neighbour proteins. **f**, The distribution of compounds against cancer-related proteins. **g**, The distribution of compounds against first neighbour proteins

Alternatively, direct targeting of central proteins is a feasible strategy in certain cases to destroy cancer cells, if the applied drug does not lead to serious side-effects.^31^ Based on the presented importance of first neighbour proteins in cancer, these, often central proteins, can also be considered as anticancer drug targets if efficient drugs (or compounds) can target them without causing major side-effects. Therefore, we investigated the currently used drugs and existing compounds with activity against cancer-related proteins and their first neighbours in the SignaLink network to point out and select potential new anti-cancer drugs (Fig. 5a). For this step we employed compound data from the ChEMBL resource,^60^ and considered a compound to be a drug if it was listed as an ‘approved drug’ according to ChEMBL (see Methods for details). To provide information on the cancer specificity of the targets, we also analysed the occurrence of cancer-related proteins and their first neighbours in different cancer types (Fig. 5b–c).

An analysis of approved drugs identified 223 drugs acting on first neighbour proteins and 122 drugs targeting cancer-related proteins (Fig. 5d–e). Importantly, from the 223 drugs against the first neighbours, only 27 (12%) have currently annotated indications in cancer, based on the anatomical therapeutic chemical^61^ (ATC) classification (Supplementary Table 8a–d). Thus, the remaining 196 drugs serve as an already existing pool for repurposing existing drugs as novel anti-cancer agents. According to our PubMed literature survey (see Methods), 83 of these 196 drugs are already under clinical trials as potential anticancer agents. 60 of these potential anticancer drugs are glucocorticoid steroids targeting the glucocorticoid receptor NR3C1, which is a first neighbour of the cancer-related protein PPARG in breast cancer and NSCLC. Pharmacological targeting of immune signal modulating proteins such as NR3C1 and PPARG were found efficient to decrease the lymphangitic metastasis formation of breast cancer and NSCLC.^62^ Another relevant example is tamoxifen, an anti-oestrogen targeting the oestrogen receptors, which are the first neighbours of the breast cancer-related HER2 (ERBB2) protein. Accordingly, tamoxifen is already being used as an endocrine modulating treatment against breast cancer.^34^

Alternatively, a source for new anticancer drugs could be compounds targeting first neighbour proteins that have not yet been applied in oncology. The number of compounds in ChEMBL^60^ against cancer-related proteins or first neighbours thereof, below a bioactivity cut-off of 500 nM, is 30 times higher than that of approved drugs against cancer-related proteins or their first neighbours (Fig. 5a; Supplementary Table 9). Thus, these compounds represent a large collection of potentially relevant agents for anticancer treatments provided they will be safe and efficient in preclinical, toxicology and clinical studies. Interestingly, the ratio between compounds targeting cancer-related or first neighbour proteins are the same as for the approved drugs (*p* > 0.05 *χ* ^2^ test; Fig. 5a). The targeting landscape of these compounds is not homogenous as we observed cancer-specific differences in their distribution (Fig. 5f,g). Remarkably, we found 675 compounds (9.9%) of the total number of compounds targeting first neighbour proteins present in all four examined cancer types. The ratio of these compounds is higher than expected (*p* < 0001 Bernoulli test to the percentage of targeted first neighbour proteins of all four cancer types), thereby giving a rationale for their experimental testing across all areas (Fig. 5g; Supplementary Table 10). To provide evidence for the applicability of some of these compounds, we checked the literature, and for those 33 compounds that have an exact name we found 10 of them are already under clinical trial against various cancer types. One such example is midostaurin, which targets multiple kinases, including MAPK9. MAPK9 is a first neighbour of a colon cancer-related protein, β-catenin, and inhibition of MAPK9 was found to down-regulate β-catenin.^63^ Supporting the probably beneficial affect of midostaurin, a previous in vitro study found that midostaurin sensitized colon cancer cells against chemotherapeutic agents.^64^

## Discussion

In this work we have shown that the first neighbours of cancer-related proteins have at least as central a position in various human signalling and PPI networks as the corresponding cancer-related proteins themselves (Fig. 1, Supplementary Fig. 1, Supplementary Table 5). Except for few direct connections, cancer-related proteins are connected through their first neighbours (Supplementary Table 3), and they can affect more Gene Ontology biological processes via their first neighbours than alone (Supplementary Table 4).

When we examined signalling systems in cancer, we found two distinct strategies how mutations and differentially expressed genes affect the network. Firstly, mutated proteins have higher or similar network centralities such as degree or betweenness, compared to those of their first neighbours. Secondly, differentially expressed proteins have lower network parameters than their first neighbours. Thus, mutated cancer-related proteins appear to exert a more direct effect onto the cellular signalling and PPI networks, whereas the differentially expressed proteins may (also) exert their effects indirectly via their first neighbours (Fig. 2). This dichotomy points out the importance of an indirect influence on proteins whose altered function is required during carcinogenesis but are too essential to be mutated (i.e., mutation of their encoding genes could decrease the viability of cancer cells). Interestingly, differentially expressed proteins can influence these central proteins in a tissue and context specific way, without interfering with all the functions of the central protein.^65^ The idea that central nodes of a network are often influenced by their neighbours is supported also by two recent studies on a fish community network and a 14 million people phone call network.^66,67^ By analysing these biological and social networks, the authors point out the role of so called influential nodes that are directly connected to high degree nodes of the network.

Current strategies in oncology mainly target mutated cancer-related proteins themselves. However, given the two strategies of cells to drive cancer elucidated in this work, we now highlighted the option to select first neighbours of differentially expressed proteins as drug targets. There are fewer drugs currently on the market, on a per protein basis, against first neighbours than against cancer-related proteins themselves; however the total number of drugs targeting first neighbours outnumbers those targeting cancer-related proteins (223 vs. 122 drugs, Fig. 5). The presented approach provides a twofold increase of testable drugs and compounds for anticancer therapy (Fig. 5a). Nonetheless, not all first neighbours can be used as drug targets, and not all drugs targeting a first neighbour could be efficient anti-cancer agents due to the complexity of the signalling network, the biochemical properties of the targets, and the highly central role of some of the first neighbours. Thus, proper selection is needed, for which we showed two complementary approaches to select the most suitable first neighbours: (1) mimicking the strategy employed by carcinogenesis and selecting those (non cancer-related) proteins that directly influence first neighbours of differentially expressed proteins having too central a position to be targeted directly, and (2) finding existing drugs and compounds targeting first neighbours in a drug repurposing setting. The two proposed strategies require further experimental analysis in a context (cancer) specific manner due to the biological complexity of both cancerous processes and signalling networks.

In our study, we examined four different types of solid cancers and employed five different and independent network resources, to show a general phenomenon. The above conclusions hold across the datasets and annotations we have used. Nevertheless, the presented study has its limitations. In particular, the definition of cancer-related proteins in this work covered mutated and differentially expressed proteins, while not taking into account copy number variation and epigenetic (e.g*.*, methylation) data. We used the widely accepted CGC as a collection of cancer type specific cancer-causing mutation; however, CGC does not contain mutations that could contribute to cancer progression without cause cancer directly.^68^ The expression data used to define differentially expressed proteins in this work were generated by microarray studies, and thus, recently produced RNAseq and protein chip datasets were not considered. This may have introduced a methodological bias. However, we think this bias would be minor, since the number of microarrays we have employed was rather large, comprising a total of 1558 arrays. To validate our external dataset based classification process, with an extensive literature search, checking 1820 papers, we analysed 82 proteins classified as first neighbours of cancer-related proteins in colon cancer, and found only 7 proteins (8.5%) that could have been classified as cancer-related if their properties have been listed correctly in the applied mutation and expression datasets (Supplementary Notes). Therefore, we consider the applied datasets well curated and their coverage appropriate for such a systems-level analysis.

Our study focused on cancer type specific carcinogenic alterations and due to the lack of sufficient amount of data, we could not take cancer heterogeneity (i.e*.*, cell-cell differences within a cancer type) into account. As for the network annotations we have used, we only considered PPI and signalling interactions, and not regulatory connections via transcription factors and microRNAs. Although these data are also available in the SignaLink 2 database,^25^ it is lacking in other network resources, thereby making the comparison of results rather difficult. Also the available cancer-specific expression datasets for miRNAs and data on active transcriptional processes are limited. Although tumour microenvironment and inter-cellular communication between different cancer cells and other cell types, such as fibroblasts are important in carcinogenesis, most of the available molecular interaction data is intracellular. Given those reasons, we in this work focused on the PPI and signalling level within a cancer cell, as here we have substantially more data from different sources available, thereby allowing us to obtain conclusions, which appear to hold more generally (and which are independent on one particular annotation).

That cancer-related proteins share significant characteristics from the network perspective has been found originally by earlier studies^10–13^ and most of those findings could be reproduced here. The only exception contrary to previous studies^69,70^ is that here cancer-related proteins did not form a connected graph. There are two explanations for this apparent discrepancy; on the one hand, we applied a much stricter definition to select cancer-related proteins, and on the other hand all of the networks we used were tissue-specific, which was not the case in previous studies. As a supporting example for the need of tissue specific studies, previously in glioblastoma first neighbours was found to act like linkers of the network allowing cancer-related proteins to affect more biological processes.^71^ We extended this observation to four other solid cancer types. The applicability of interaction data to extend the set of disease-related genes was already successfully applied in previous studies.^14,15^ A recent analysis focusing on the network modules of disease-related genes (diseasomes) identified potential disease genes by using various network data.^69^ Compared to this diseaseome study,^69^ our approach focused only on the direct physical interactors of cancer-related proteins, and did not extend the scope based on regulatory connections and the module structure of the network. By selecting specific interaction data and simpler measurements of the network, the presented study straightforwardly points out cancer-specific key proteins, not listed before based solely on mutation and differential expression screens.

In the field of graph theory, it is known that randomly selecting nodes in a graph and then looking for their first neighbours result in identifying high degree and high betweenness nodes.^72^ Accordingly, we found the same in our study by selecting cancer-related proteins and checking the network parameters of their first neighbours (Supplementary Table 5). Interestingly, most of these first neighbours were also found in a randomization test when we randomly selected a set of proteins and looked for their first neighbours (Supplementary Fig. 3). Therefore, to classify a protein as a first neighbour is independent of the original list of cancer-related proteins, and depends mostly on the network topology. Surprisingly, this phenomenon has never been analysed and presented before as a systems-level feature of carcinogenesis. Thus, in the current study we connected a general graph-theory phenomena with actual cancer and drug discovery related problems.

In conclusion, with five different networks and in four cancer types we have shown that first neighbour proteins are at least as central locally and globally (i.e.*,* have similar degree and betweenness) as the cancer-related proteins themselves. While mutated proteins in central positions may have a more direct effect on the cellular network, differentially expressed proteins, which mostly localize to less central positions but often next to a major protein, appear to impact the network more extensively via these first neighbours. This observation opens up new strategies for target selection, and hence, anticancer drug discovery.

## Methods

### Cancer type

We selected four different solid cancer types (colon, non-small cell lung, breast and hepatocellular carcinoma). As our aim was to indicate general tissue specific carcinogenic properties, we used all the known subtypes of these cancers, including (1) hyper mutated and non-hypermutated colon cancers; (2) lung adenocarcinomas, large cell carcinomas, and squamous cell carcinomas (3) HER2 positive, basal, luminal A and luminal B breast cancers; and (4) hepatitis B or hepatitis C, cirrhosis or other agent causing hepatocellular carcinoma data. We did not distinguish between left sided and right sided colon cancer to provide a more general feature for this cancer type.

### Mutation data

The CGC,^5^ database was used to retrieve tissue specific mutation data of the four different kind cancer we used (Downloaded: 15 January 2015). The keywords we searched for are listed in the Supplementary Table 1 ordered by cancer type. Entrez genes were mapped to Uniprot accessions (ACs) with the UniProt mapping service.^73^

### Microarray data

For the expression data, we extracted all available Affymetrix HGU133 plus2 chip microarray datasets from GEO,^24^ web resource, for the four selected cancer types, if normal tissue controls were also available (downloaded in August, 2014). The microarray studies we used for this study are listed in Supplementary Table 11. We renormalized the chip reads with the Robust multi-array average method.^74^ All chips were normalized to each other. After that, the probe sets were matched to UniProt ACs, using the probe set showing the highest level of expression in case more than one mapped to the same ID. We used these expression values to determine the expressed genes for each network in each normal tissue and each cancer type.

### Network resources

We used three signalling network resources for the analysis: the SignaLink 2 signalling network resource,^25^ the Reactome database,^26^ and the signalling network from the study by Cui *et al*. 2007.^10^ In addition, we used two PPI networks: The manually curated HPRD database,^27^ and a combined network of a more diverse and more up-to-date set of resources: IntAct,^3^ DIP,^28^ and BioGRID.^30^ For SignaLink 2, where predicted and integrated information is also available, we used only the manually curated pathway data, which is fully independent from the other sources. All databases were downloaded on 27 January 2015. We mapped the protein identifiers to UniProt ACs with the Uniprot mapping service.^73^ It allowed the merging of the three resources. We used only reviewed UniProt ACs (Swissprot). If there was multiple Swissprot ACs for one protein, we kept them all. Proteins that were not mapped to such an accession have been discarded. We have not used other big integrated PPI resources, such as STRING, as most of the resources we used here are present in STRING as well.^75^ Thus, analyzing the different sources separately provides more information on the different origin of data.

### Tissue and cancer-specific networks

Using the collected expression data, and the different signalling and PPI databases, we constructed the tissue specific healthy and cancerous signalling and PPI networks. After calculating the mean and standard deviation of expression per cancer, we defined that proteins are considered not expressed if their mRNA expression levels were below the mean minus the standard deviation in the given network. We considered a protein differentially expressed in a given tissue, if the corresponding mRNA was either present only in normal tissue and absent in cancerous tissue, or other way around. Thus, simple overexpression of otherwise normally expressed genes were not considered to select only the outstanding expressional differences. In this way, our analysis was more sensitive to the genes having lower expression. Interactions between proteins were considered if both interacting proteins were present in either healthy or cancerous tissue. In this way we got a tissue-specific network, where we can see the network effect of cancer-related differently expressed proteins.

### First neighbour proteins

First neighbours of cancer-related proteins were defined as proteins (1) directly and physically interacting with cancer-related proteins (according the network databases used, see below), and (2) which were not cancer-related protein themselves. Thus, if a cancer-related protein was also first neighbour of another cancer-related protein it was considered only as cancer-related protein to avoid the overlap and allow the analysis of the clear first neighbours that have not been related to the given cancer before.

### Gene ontology analysis

The gene ontology (GO) analysis aim was to determine, whether the cancer related proteins could reach more processes with their first neighbours, than expected. Gene Ontology information (gene_association.goa_human) was downloaded from Gene Ontology website (on 2 June 2015). Only biological processes (BP) were considered. The cancer-related proteins were annotated first, then the first neighbours, which got only those GO BPs that were not annotated before to a cancer protein. This way we could focus on the added functions from first neighbour proteins. After that, we measured with Binomial test whether first neighbours have more GO BPs than expected based on their ratio from the network. This approach is stricter than considering all GO BPs that were annotated to both cancer-related and first neighbour proteins.

### Giant component analysis

We used exact statistics to determine the giant components percentage. We perturbed the nodes annotation 1000 times and calculated the giant component ratio. After that we used a *Z* score based outlier statistical test to determine the data’s significance.

### Network topology parameters

We measured three major network parameters for each node (proteins): (1) degree, which is the number of its interactions; (2) betweenness centrality, which is another importance measure that is equal to the number of shortest paths from all nodes to all others that pass through the node of interest, and (3) clustering coefficient, which measures how the neighbours of the node of interest are also connected to each other (form a cluster).

We used the Igraph,^76^ Python plugin to calculate the parameters for each network. Edges in all networks are presented without weight, and self-loops have been removed. Though some of the signalling networks contained direction for an interaction, due to the lack of general comparisons, we measured the network parameters without taking direction into account. We measured the parameters in the non-tissue specific, original networks as well.

### Randomization analysis

We investigated that the first neighbour proteins central role remains if we randomly selected proteins for our analyses. For 100 times, in the cancer type specific networks of SignaLink, we randomly selected the same number of proteins as the original set of cancer-related proteins contained. Then, for all 100 cases in each cancer type, we listed the first neighbour interactors of the randomly selected proteins. Finally, we measured the occurrence of each protein in the network as first neighbour and compared this list with the previously identified, “real” list of first neighbours.

### Drug and compound analysis

Data on drugs and compounds were downloaded from the ChEMBL Database^77^ version 20. We considered a compound as drug if it had been subjected to Phase 4 clinical tests according to ChEMBL. We used those compounds, which are targeting proteins in SignaLink database. We have implemented a relatively strict bioactivity cut-off of 500nM (IC50, Ki, Kd) during our filtering process to identify these compounds, which indicates good activity with a strong potential for therapeutic applications. Importantly, these compounds could provide opportunities for structure/scaffold similarity studies to identify a recurring sub-structure, the knowledge from which could be utilized in structure/fragment-based drug designing studies. Drug indications have been classified according to the ATC database.^61^

### Literature mining methods

We used Biopython^78^ to query PubMed and download the abstracts of the articles. We searched for the drug or compound name (used in ChEMBL) plus “cancer” and “treatment” as further keywords. We limited our PubMed search for clinical trials.

### Statistics

Wilcoxon rank-sum test and Kolmogorov-Smirnov tests have been conducted for nonparametric hypothesis testing. Throughout the text, only Wilcoxon rank-sum test results were presented, as Kolmogorov–Smirnov tests results have appeared to be concordant. The results of all statistics can be found in the supplementary tables. The whole network was used as control, except when noted. We used Binomial tests to compare to a given percentage. If it is not mentioned otherwise, the compared ratio is first neighbour proteins to all proteins. We used the Numpy and SciPy packages for Python for statistical analysis.^79^

### Other programs

For creating the network figures, version 3.1 of Cytoscape^80^ was used. Violin plots have been made with the vioplot R package,^81^ while supplementary boxplots have been constructed with the help of the matplotlib python package.^82^

## Electronic supplementary material

Supplementary Information

Supplementary Fig. 1

Supplementary Fig. 2

Supplementary Fig. 3

Supplementary Fig. 4

Supplementary Fig. 5

Supplementary Fig. 6

Supplementary Table 1

Supplementary Table 2

Supplementary Table 3

Supplementary Table 4

Supplementary Table 5

Supplementary Table 6

Supplementary Table 7

Supplementary Table 8

Supplementary Table 9

Supplementary Table 10

Supplementary Table 11
