# Supplementary material for: Neighbours of cancer-related proteins have key influence on pathogenesis and could increase the drug target space for anticancer therapies
Source: NPJ Syst Biol Appl. 2017 Jan 24;3:2. doi: 10.1038/s41540-017-0003-6 (PMC5460138; doi:10.1038/s41540-017-0003-6)
Supplement: Supplementary file 1 — Supplementary Information [file 41540_2017_3_MOESM1_ESM.docx]

**Neighbours of cancer-related proteins have key influence
on pathogenesis and could increase the drug target space for anti-cancer therapies**

Dezső Módos, Krishna C. Bulusu, Dávid Fazekas, , János Kubisch, Johanne Brooks, István Marcell, Péter M. Szabó, Tibor Vellai, Péter Csermely, Katalin Lenti, Andreas Bender, Tamás Korcsmáros

# **Supplementary Notes**

**Manual check of the first neighbour classification**

To control our methods, we searched for scientific publications about 82 proteins found and classified by our described method as first neighbours of differentially expressed proteins in colon cancer. We used this subset of first neighbour proteins as even this list resulted in 1820 publications to check. We manually checked the abstracts of all the 1820 papers. With this extensive search, we found that 38 out of the 82 proteins (46%) have been implicated in carcinogenesis. We used this set as a control to the original mutation and expression datasets we applied in our classification process (see Supplementary Methods). Detailed analysis showed that only four of them have been described as their mutation could cause colon cancer, and only three of them were shown with high sample count that they have a differential expression in colon cancer. Thus, we concluded that only 7 out of the 82 proteins (8.5%) could be considered as false positives as clear first neighbour proteins (i.e., proteins found as direct interactors of cancer-related proteins but not listed as mutated or differentially expressed proteins in the applied datasets), and they should have been listed as cancer-related proteins. Among the 82 proteins, we also found 11 proteins having single nucleotide polymorphisms, which increase cancer severity or risk of colon cancer, and 17 proteins whose expression is associated with colon cancer severity or metastasis susceptibility. None of these properties were used when we defined cancer-related proteins. Therefore, our extensive manual curation pointed out that 91.5% of the first neighbours classified by our method were accurate according to the literature as well.

**References**

1. Berg, C. *et al.* *Guidelines for ATC classification and DDD assignment 2015*. (WHO Collaborating Centre for Drug Statistics Methodology Norwegian Institute of Public Health, 2014). at <http://www.whocc.no/filearchive/publications/2015_guidelines.pdf>

2. Bento, A. P. *et al.* The ChEMBL bioactivity database: an update. *Nucleic Acids Res.* **42,** D1083–90 (2014).

3. Siegel, R. L., Miller, K. D. & Jemal, A. Cancer Statistics, 2015. *CA Cancer J Clin* **65,** 5–29 (2015).
