# Supplementary material for: Neighbours of cancer-related proteins have key influence on pathogenesis and could increase the drug target space for anticancer therapies
Source: NPJ Syst Biol Appl. 2017 Jan 24;3:2. doi: 10.1038/s41540-017-0003-6 (PMC5460138; doi:10.1038/s41540-017-0003-6)
Supplement: Supplementary file 2 — Supplementary Fig. 1 [file 41540_2017_3_MOESM2_ESM.pptx]

## Slide 1
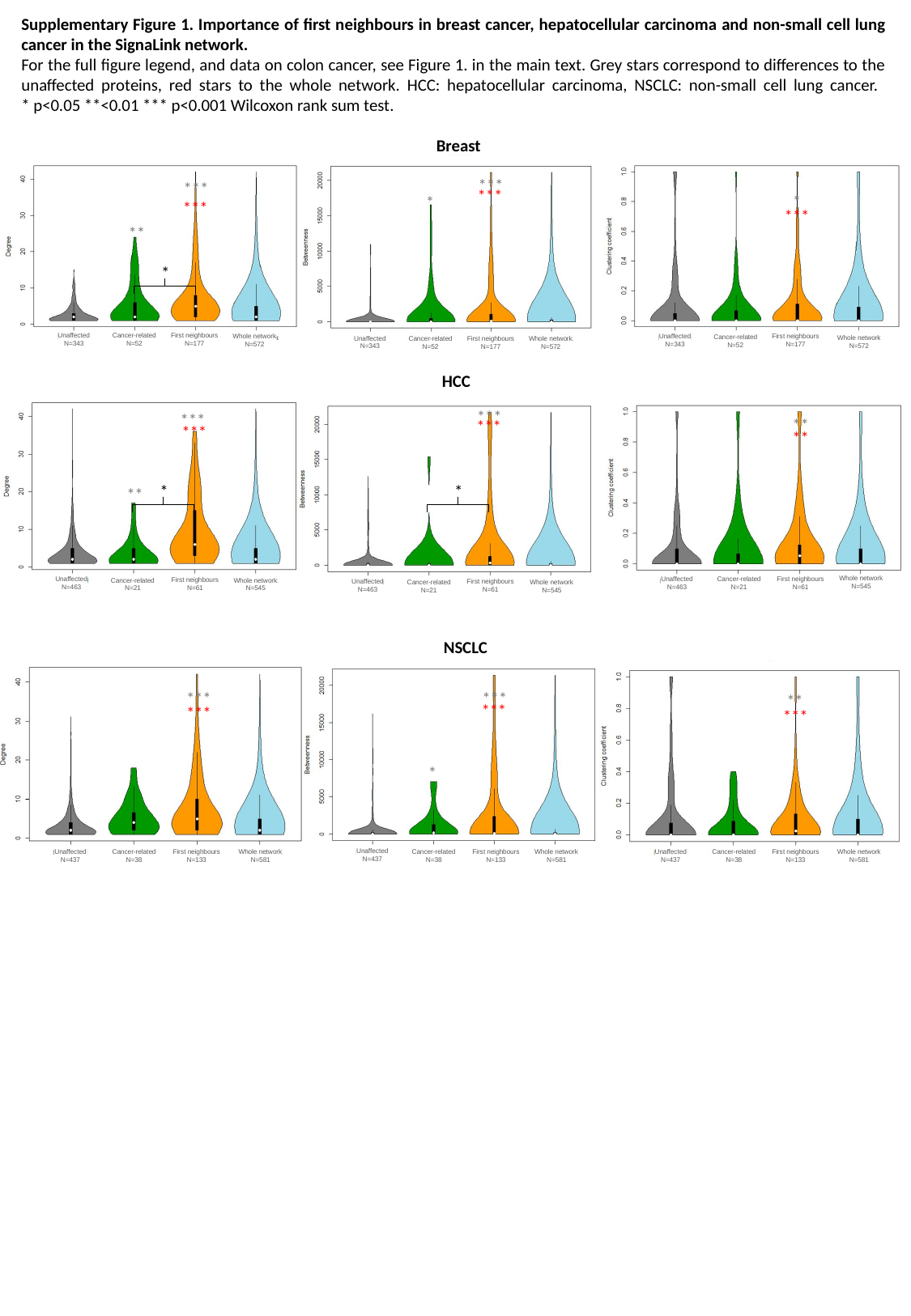

Supplementary Figure 1. Importance of first neighbours in breast cancer, hepatocellular carcinoma and non-small cell lung cancer in the SignaLink network.
For the full figure legend, and data on colon cancer, see Figure 1. in the main text. Grey stars correspond to differences to the unaffected proteins, red stars to the whole network. HCC: hepatocellular carcinoma, NSCLC: non-small cell lung cancer.
* p<0.05 **<0.01 *** p<0.001 Wilcoxon rank sum test.
Breast
***
***
***
*
*
***
***
**
*
Unaffected
N=343
First neighbours
N=177
Cancer-related
N=52
Whole network
N=572
Unaffected
N=343
First neighbours
N=177
Cancer-related
N=52
Whole network
N=572
Unaffected
N=343
First neighbours
N=177
Cancer-related
N=52
Whole network
N=572
HCC
***
***
**
***
***
**
*
*
**
Whole network
N=545
Cancer-related
N=21
First neighbours
N=61
Unaffected
N=463
Unaffected
N=463
First neighbours
N=61
Cancer-related
N=21
Whole network
N=545
Unaffected
N=463
First neighbours
N=61
Cancer-related
N=21
Whole network
N=545
NSCLC
***
***
**
***
***
***
*
Unaffected
N=437
Unaffected
N=437
First neighbours
N=133
Cancer-related
N=38
First neighbours
N=133
Whole network
N=581
Cancer-related
N=38
Whole network
N=581
Unaffected
N=437
Cancer-related
N=38
First neighbours
N=133
Whole network
N=581
