# Supplementary material for: Neighbours of cancer-related proteins have key influence on pathogenesis and could increase the drug target space for anticancer therapies
Source: NPJ Syst Biol Appl. 2017 Jan 24;3:2. doi: 10.1038/s41540-017-0003-6 (PMC5460138; doi:10.1038/s41540-017-0003-6)
Supplement: Supplementary file 3 — Supplementary Fig. 2 [file 41540_2017_3_MOESM3_ESM.pptx]

## Slide 1
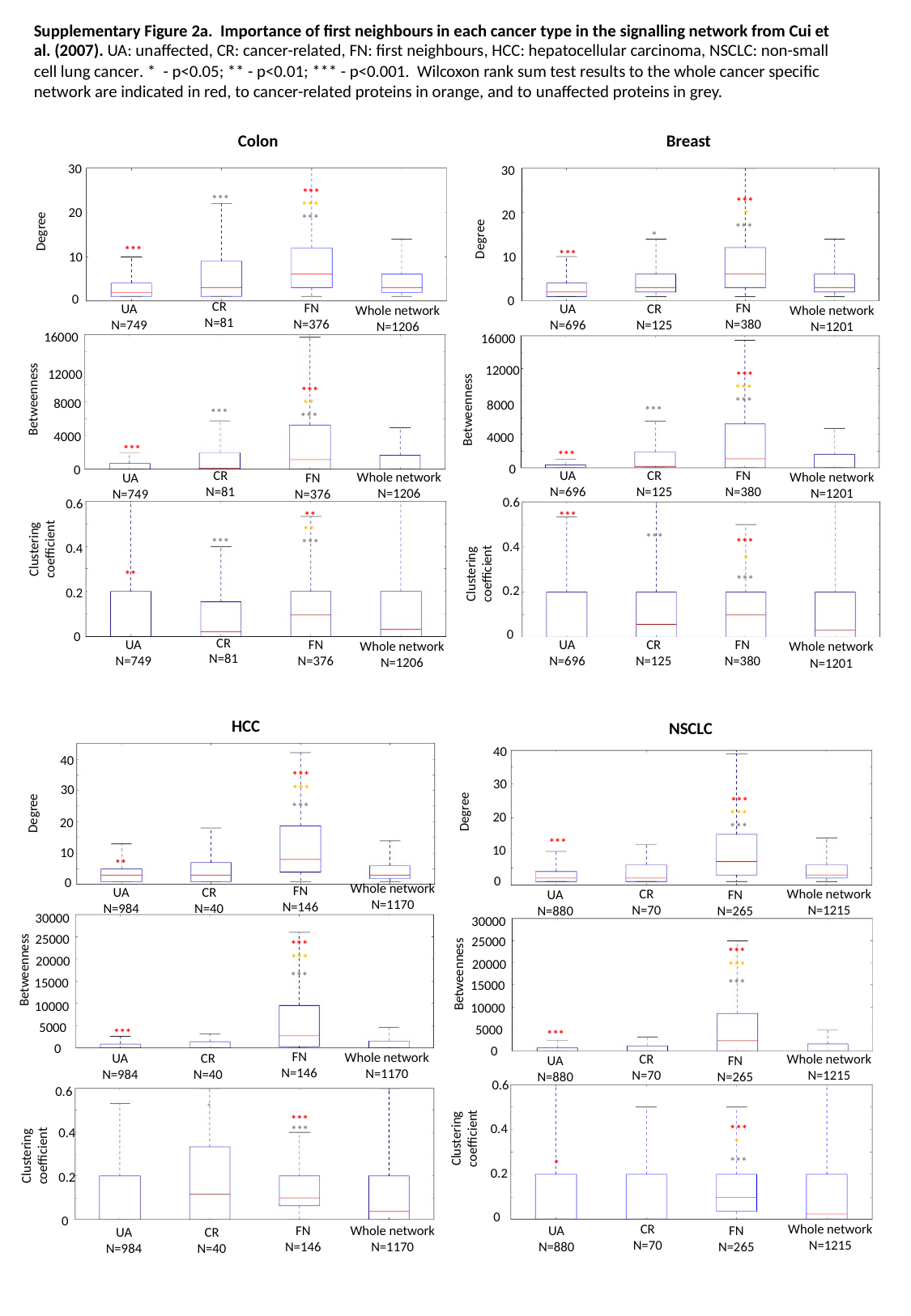

Supplementary Figure 2a. Importance of first neighbours in each cancer type in the signalling network from Cui et al. (2007). UA: unaffected, CR: cancer-related, FN: first neighbours, HCC: hepatocellular carcinoma, NSCLC: non-small cell lung cancer. * - p<0.05; ** - p<0.01; *** - p<0.001. Wilcoxon rank sum test results to the whole cancer specific network are indicated in red, to cancer-related proteins in orange, and to unaffected proteins in grey.
Breast
Colon
30
30
***
***
***
***
20
20
*
***
***
Degree
*
Degree
***
***
10
10
0
0
CR
N=81
FN
N=380
FN
N=376
UA
N=749
CR
N=125
UA
N=696
Whole network
N=1201
Whole network
N=1206
16000
16000
12000
12000
***
***
***
Betweenness
***
8000
8000
**
Betweenness
***
***
***
4000
4000
***
***
0
0
FN
N=380
CR
N=125
UA
N=696
CR
N=81
Whole network
N=1206
Whole network
N=1201
FN
N=376
UA
N=749
0.6
0.6
**
***
**
***
Clustering coefficient
***
***
***
0.4
0.4
*
Clustering coefficient
**
***
0.2
0.2
0
0
CR
N=81
FN
N=376
FN
N=380
UA
N=749
CR
N=125
UA
N=696
Whole network
N=1206
Whole network
N=1201
HCC
NSCLC
40
40
***
30
30
***
***
***
Degree
Degree
***
20
20
***
***
10
10
**
0
0
Whole network
N=1170
FN
N=146
UA
N=984
CR
N=40
CR
N=70
Whole network
N=1215
UA
N=880
FN
N=265
30000
30000
25000
25000
***
***
***
20000
20000
***
Betweenness
Betweenness
***
15000
***
15000
10000
10000
5000
5000
***
***
0
0
FN
N=146
Whole network
N=1170
UA
N=984
CR
N=40
CR
N=70
Whole network
N=1215
UA
N=880
FN
N=265
0.6
0.6
*
***
0.4
***
Clustering coefficient
***
0.4
*
Clustering coefficient
***
*
0.2
0.2
0
0
CR
N=70
Whole network
N=1215
FN
N=146
Whole network
N=1170
UA
N=880
FN
N=265
UA
N=984
CR
N=40

## Slide 2
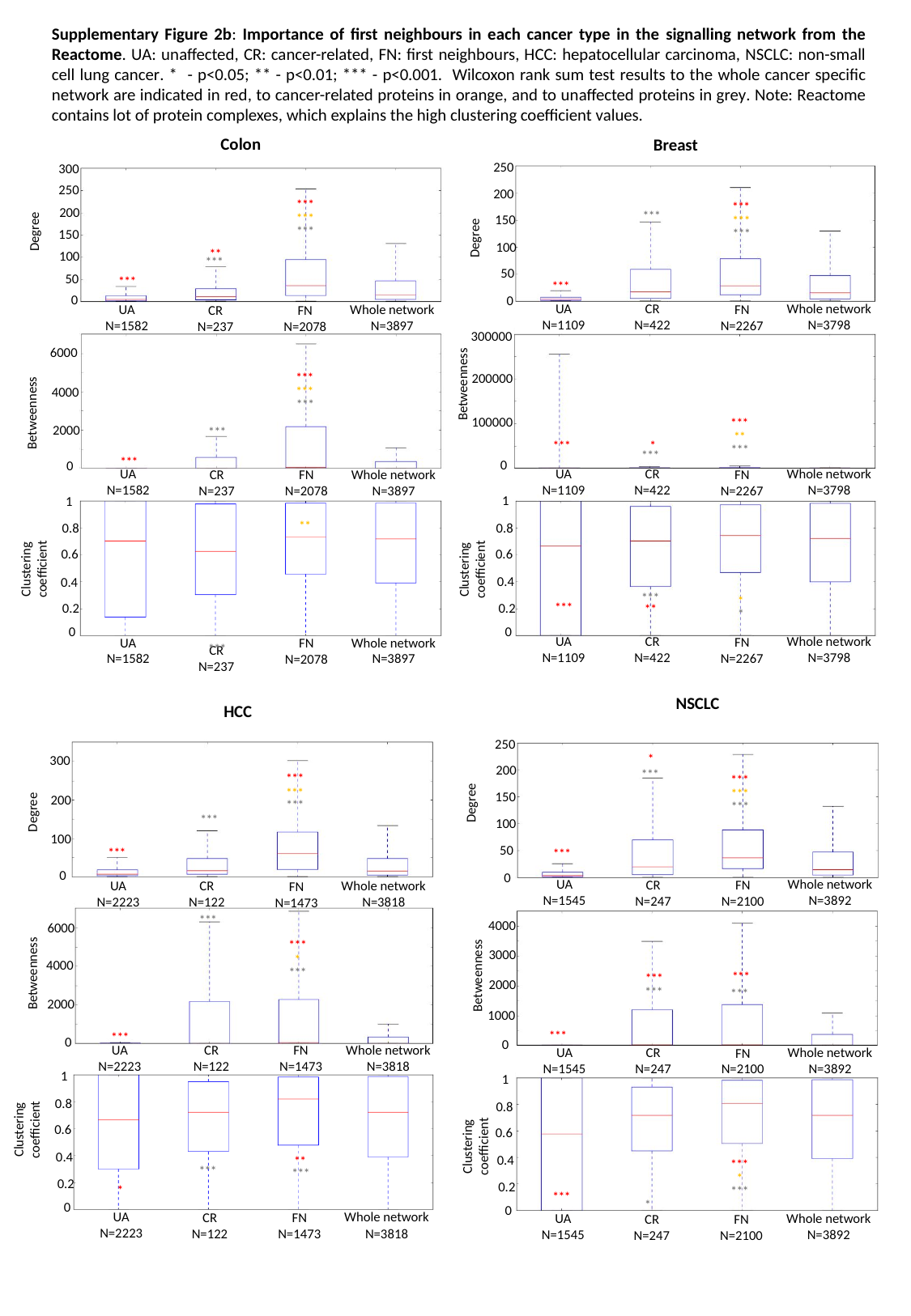

Supplementary Figure 2b: Importance of first neighbours in each cancer type in the signalling network from the Reactome. UA: unaffected, CR: cancer-related, FN: first neighbours, HCC: hepatocellular carcinoma, NSCLC: non-small cell lung cancer. * - p<0.05; ** - p<0.01; *** - p<0.001. Wilcoxon rank sum test results to the whole cancer specific network are indicated in red, to cancer-related proteins in orange, and to unaffected proteins in grey. Note: Reactome contains lot of protein complexes, which explains the high clustering coefficient values.
Colon
Breast
250
300
250
200
***
***
200
***
***
150
***
Degree
***
***
150
Degree
100
**
100
***
50
50
***
***
0
0
UA
N=1109
Whole network
N=3798
CR
N=422
FN
N=2267
UA
N=1582
Whole network
N=3897
CR
N=237
FN
N=2078
300000
6000
***
200000
Betweenness
4000
***
***
Betweenness
100000
***
2000
***
**
*
***
***
***
***
0
0
UA
N=1109
Whole network
N=3798
CR
N=422
UA
N=1582
FN
N=2267
Whole network
N=3897
CR
N=237
FN
N=2078
1
1
**
0.8
0.8
0.6
0.6
Clustering coefficient
Clustering coefficient
0.4
0.4
***
*
0.2
***
0.2
**
*
0
0
UA
N=1109
Whole network
N=3798
CR
N=422
FN
N=2267
UA
N=1582
Whole network
N=3897
FN
N=2078
***
CR
N=237
NSCLC
HCC
250
*
300
200
***
***
***
***
***
150
200
Degree
***
***
Degree
***
100
100
50
***
***
0
0
UA
N=1545
Whole network
N=3892
CR
N=247
FN
N=2100
UA
N=2223
Whole network
N=3818
CR
N=122
FN
N=1473
***
4000
6000
***
3000
*
4000
***
Betweenness
Betweenness
***
***
2000
***
***
2000
1000
***
***
0
0
Whole network
N=3818
CR
N=122
UA
N=2223
FN
N=1473
UA
N=1545
Whole network
N=3892
CR
N=247
FN
N=2100
1
1
0.8
0.8
Clustering coefficient
0.6
0.6
Clustering coefficient
0.4
0.4
**
***
***
***
*
0.2
0.2
*
***
***
*
0
0
UA
N=2223
Whole network
N=3818
CR
N=122
FN
N=1473
UA
N=1545
Whole network
N=3892
CR
N=247
FN
N=2100

## Slide 3
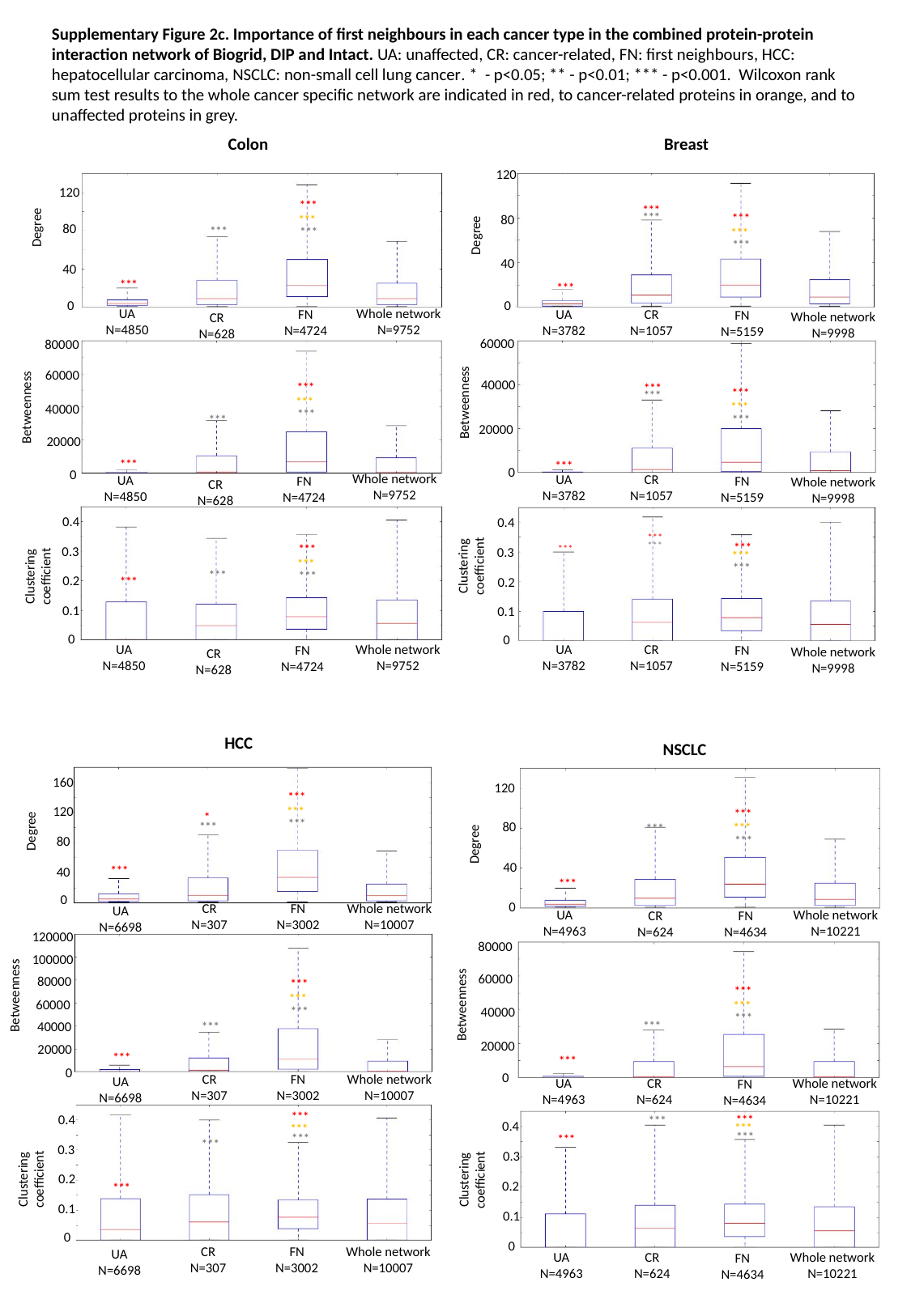

Supplementary Figure 2c. Importance of first neighbours in each cancer type in the combined protein-protein interaction network of Biogrid, DIP and Intact. UA: unaffected, CR: cancer-related, FN: first neighbours, HCC: hepatocellular carcinoma, NSCLC: non-small cell lung cancer. * - p<0.05; ** - p<0.01; *** - p<0.001. Wilcoxon rank sum test results to the whole cancer specific network are indicated in red, to cancer-related proteins in orange, and to unaffected proteins in grey.
Breast
Colon
120
120
***
***
***
***
80
***
Degree
80
***
***
***
Degree
***
40
40
***
***
0
0
UA
N=4850
Whole network
N=9752
FN
N=4724
CR
N=1057
UA
N=3782
FN
N=5159
Whole network
N=9998
CR
N=628
60000
80000
60000
40000
***
***
***
***
***
Betweenness
Betweenness
***
40000
***
***
***
20000
20000
***
***
0
0
Whole network
N=9752
CR
N=1057
UA
N=3782
UA
N=4850
FN
N=5159
FN
N=4724
Whole network
N=9998
CR
N=628
0.4
0.4
***
***
***
***
***
0.3
0.3
***
Clustering coefficient
***
***
Clustering coefficient
***
***
0.2
0.2
***
0.1
0.1
0
0
Whole network
N=9752
CR
N=1057
UA
N=3782
UA
N=4850
FN
N=4724
FN
N=5159
Whole network
N=9998
CR
N=628
HCC
NSCLC
160
120
***
120
***
***
*
***
80
***
***
***
Degree
80
***
Degree
40
***
40
***
0
0
CR
N=307
FN
N=3002
Whole network
N=10007
UA
N=6698
UA
N=4963
Whole network
N=10221
CR
N=624
FN
N=4634
120000
80000
100000
60000
80000
***
***
Betweenness
***
60000
Betweenness
***
***
40000
***
***
40000
***
20000
20000
***
***
0
0
CR
N=307
FN
N=3002
Whole network
N=10007
UA
N=6698
UA
N=4963
Whole network
N=10221
CR
N=624
FN
N=4634
***
0.4
***
***
0.4
***
***
***
***
***
***
0.3
0.3
Clustering coefficient
Clustering coefficient
0.2
0.2
***
0.1
0.1
0
0
CR
N=307
FN
N=3002
Whole network
N=10007
UA
N=6698
UA
N=4963
Whole network
N=10221
CR
N=624
FN
N=4634

## Slide 4
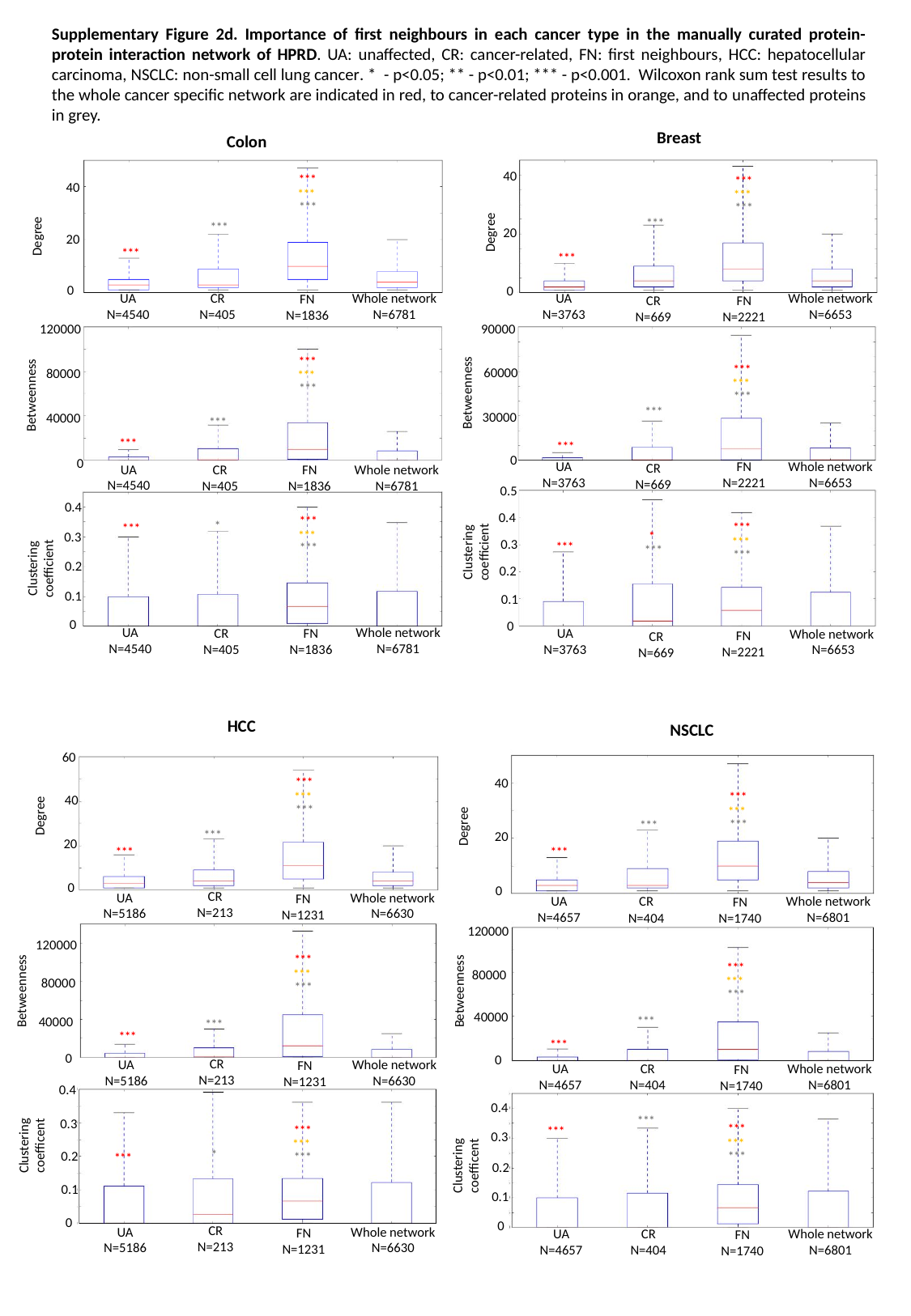

Supplementary Figure 2d. Importance of first neighbours in each cancer type in the manually curated protein-protein interaction network of HPRD. UA: unaffected, CR: cancer-related, FN: first neighbours, HCC: hepatocellular carcinoma, NSCLC: non-small cell lung cancer. * - p<0.05; ** - p<0.01; *** - p<0.001. Wilcoxon rank sum test results to the whole cancer specific network are indicated in red, to cancer-related proteins in orange, and to unaffected proteins in grey.
Breast
Colon
40
***
***
40
***
***
***
***
***
***
20
Degree
Degree
20
***
***
0
0
UA
N=4540
UA
N=3763
Whole network
N=6653
Whole network
N=6781
CR
N=405
FN
N=1836
FN
N=2221
CR
N=669
90000
120000
***
***
60000
80000
***
***
***
Betweenness
Betweenness
***
***
30000
40000
***
***
***
0
0
FN
N=2221
UA
N=3763
Whole network
N=6653
CR
N=669
UA
N=4540
Whole network
N=6781
CR
N=405
FN
N=1836
0.5
0.4
0.4
***
*
***
***
***
*
0.3
***
0.3
Clustering coefficient
***
***
***
***
Clustering coefficient
0.2
0.2
0.1
0.1
0
0
UA
N=4540
Whole network
N=6781
CR
N=405
FN
N=1836
UA
N=3763
Whole network
N=6653
FN
N=2221
CR
N=669
HCC
NSCLC
60
***
40
***
***
40
***
***
Degree
***
***
Degree
***
20
20
***
***
0
0
CR
N=213
UA
N=5186
Whole network
N=6630
FN
N=1231
UA
N=4657
Whole network
N=6801
CR
N=404
FN
N=1740
120000
120000
***
***
80000
***
***
80000
***
Betweenness
Betweenness
***
40000
40000
***
***
***
***
0
0
CR
N=213
UA
N=5186
Whole network
N=6630
FN
N=1231
UA
N=4657
Whole network
N=6801
CR
N=404
FN
N=1740
0.4
0.4
***
0.3
***
***
***
0.3
Clustering coefficent
***
***
*
0.2
***
Clustering coefficent
***
***
0.2
0.1
0.1
0
0
CR
N=213
UA
N=5186
Whole network
N=6630
FN
N=1231
UA
N=4657
Whole network
N=6801
CR
N=404
FN
N=1740
