# Supplementary material for: Neighbours of cancer-related proteins have key influence on pathogenesis and could increase the drug target space for anticancer therapies
Source: NPJ Syst Biol Appl. 2017 Jan 24;3:2. doi: 10.1038/s41540-017-0003-6 (PMC5460138; doi:10.1038/s41540-017-0003-6)
Supplement: Supplementary file 4 — Supplementary Fig. 3 [file 41540_2017_3_MOESM4_ESM.pptx]

## Slide 1
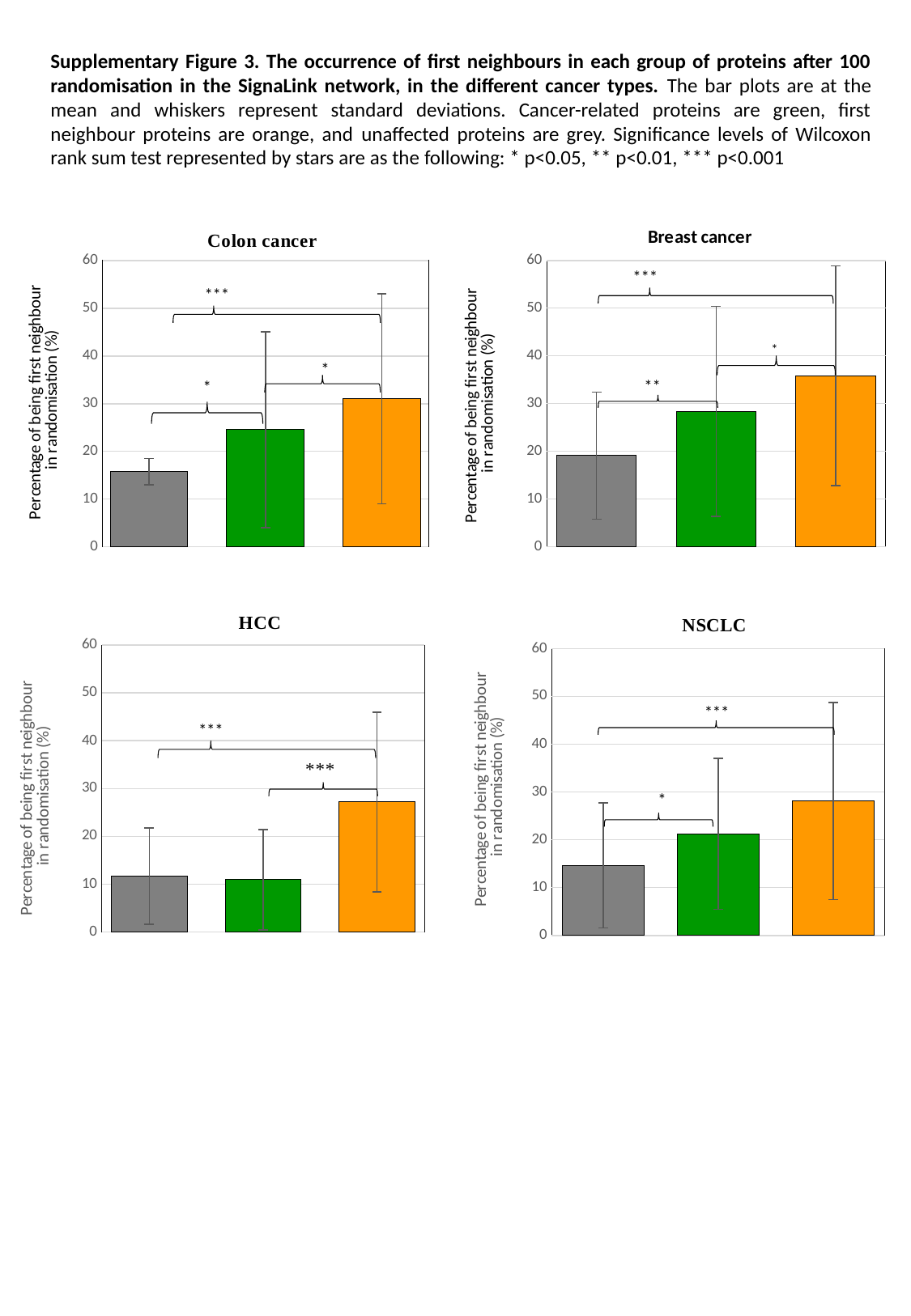

Supplementary Figure 3. The occurrence of first neighbours in each group of proteins after 100 randomisation in the SignaLink network, in the different cancer types. The bar plots are at the mean and whiskers represent standard deviations. Cancer-related proteins are green, first neighbour proteins are orange, and unaffected proteins are grey. Significance levels of Wilcoxon rank sum test represented by stars are as the following: * p<0.05, ** p<0.01, *** p<0.001
### Chart
| Category | Not affected | Cancer realted | First neighbours |
|---|---|---|---|
| Colon | 15.7543 | 24.5526 | 31.0226 |
### Chart
| Category | Not affected | Cancer realted | First neighbours |
|---|---|---|---|
| Breast | 19.0816 | 28.3462 | 35.79660000000001 |***
***
*
*
**
*
### Chart
| Category | Not affected | Cancer realted | First neighbours |
|---|---|---|---|
| Liver | 11.668 | 10.9091 | 27.1765 |
### Chart
| Category | Not affected | Cancer realted | First neighbours |
|---|---|---|---|
| Lung | 14.6636 | 21.2581 | 28.115 |
***
***
*
