# Supplementary material for: Neighbours of cancer-related proteins have key influence on pathogenesis and could increase the drug target space for anticancer therapies
Source: NPJ Syst Biol Appl. 2017 Jan 24;3:2. doi: 10.1038/s41540-017-0003-6 (PMC5460138; doi:10.1038/s41540-017-0003-6)
Supplement: Supplementary file 5 — Supplementary Fig. 4 [file 41540_2017_3_MOESM5_ESM.pptx]

## Slide 1
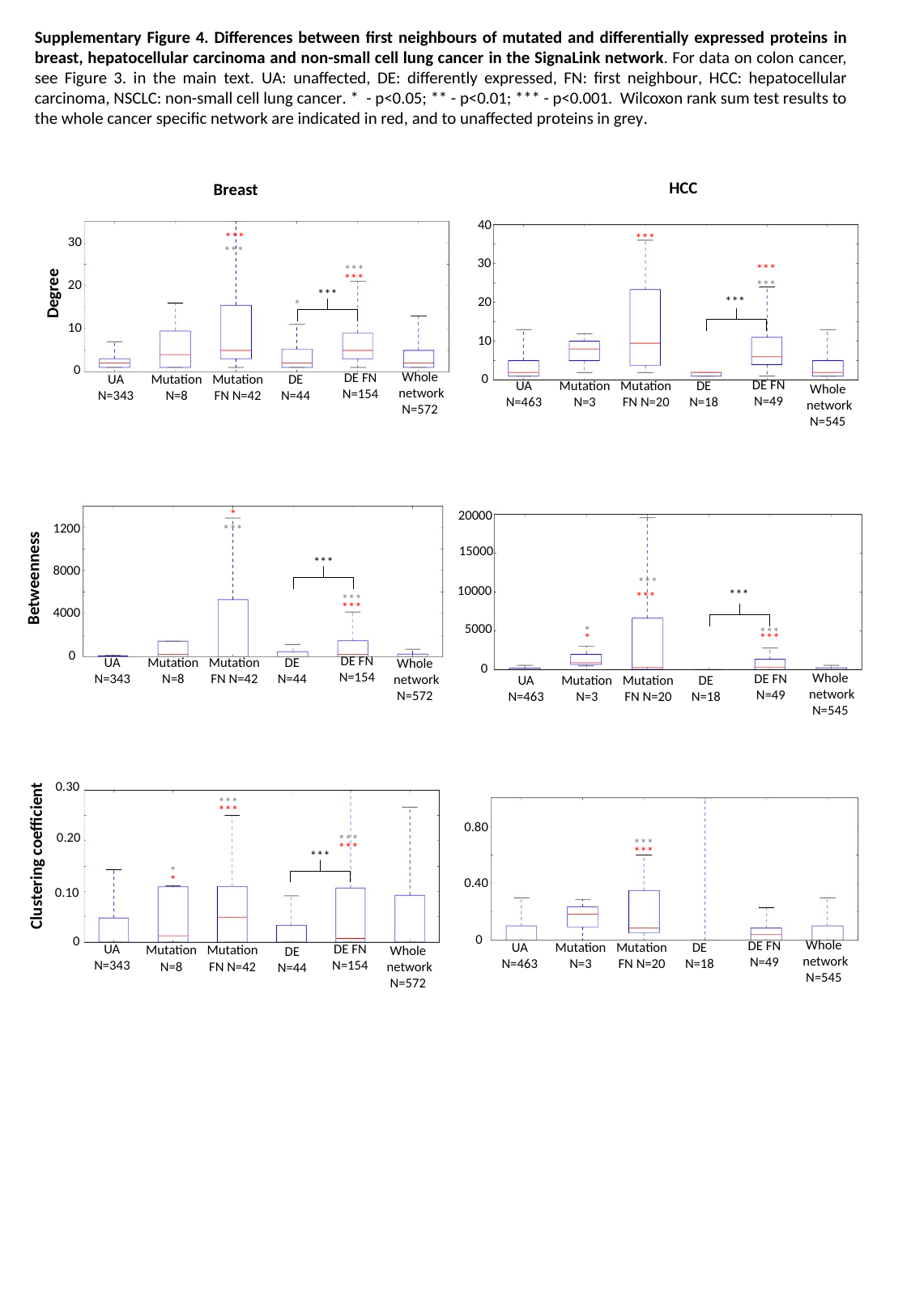

Supplementary Figure 4. Differences between first neighbours of mutated and differentially expressed proteins in breast, hepatocellular carcinoma and non-small cell lung cancer in the SignaLink network. For data on colon cancer, see Figure 3. in the main text. UA: unaffected, DE: differently expressed, FN: first neighbour, HCC: hepatocellular carcinoma, NSCLC: non-small cell lung cancer. * - p<0.05; ** - p<0.01; *** - p<0.001. Wilcoxon rank sum test results to the whole cancer specific network are indicated in red, and to unaffected proteins in grey.
HCC
Breast
40
***
***
30
***
30
***
***
***
***
20
Degree
***
20
***
*
10
10
0
Whole
 network
N=572
DE FN
N=154
0
UA
N=343
Mutation
N=8
DE
N=44
Mutation
FN N=42
DE FN
N=49
UA
N=463
Mutation
N=3
DE
N=18
Mutation
FN N=20
Whole
 network
N=545
*
20000
1200
***
15000
***
8000
Betweenness
***
10000
***
***
***
***
4000
5000
*
***
*
***
0
DE FN
N=154
UA
N=343
Mutation
N=8
DE
N=44
Mutation
FN N=42
Whole
 network
N=572
0
Whole
 network
N=545
DE FN
N=49
UA
N=463
Mutation
N=3
DE
N=18
Mutation
FN N=20
0.30
***
***
0.80
0.20
***
***
***
***
Clustering coefficient
***
*
*
0.40
0.10
0
0
Whole
 network
N=545
DE FN
N=49
UA
N=463
Mutation
N=3
DE
N=18
Mutation
FN N=20
DE FN
N=154
UA
N=343
Mutation
N=8
Mutation
FN N=42
Whole
 network
N=572
DE
N=44

## Slide 2
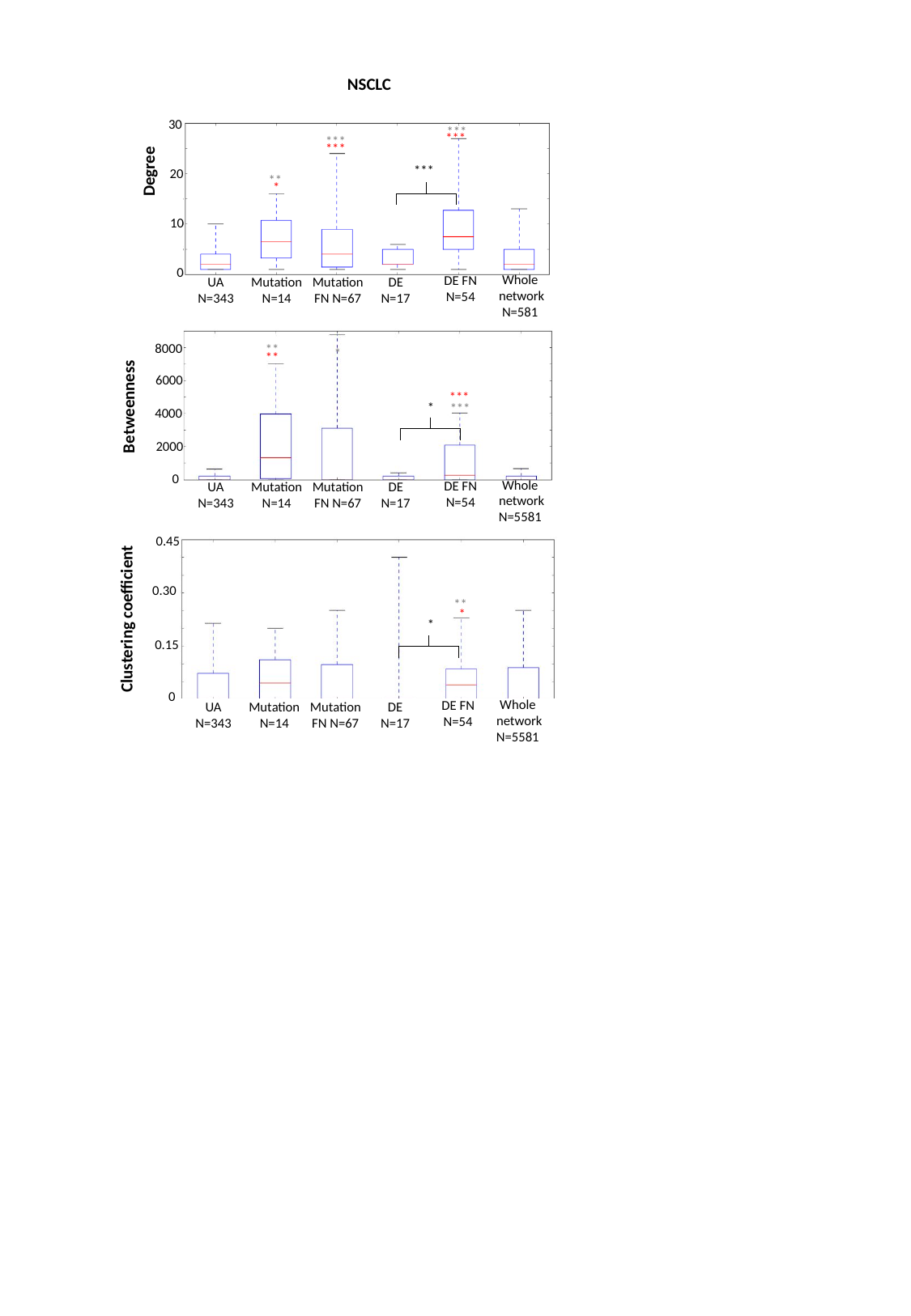

NSCLC
30
***
***
***
***
Degree
***
20
**
*
10
0
Whole
 network
N=581
DE FN
N=54
UA
N=343
Mutation
N=14
DE
N=17
Mutation
FN N=67
8000
**
*
**
6000
***
Betweenness
*
***
4000
2000
0
Whole
 network
N=5581
DE FN
N=54
UA
N=343
Mutation
N=14
DE
N=17
Mutation
FN N=67
0.45
0.30
**
*
Clustering coefficient
*
0.15
0
Whole
 network
N=5581
DE FN
N=54
UA
N=343
DE
N=17
Mutation
N=14
Mutation
FN N=67
