# Supplementary material for: Neighbours of cancer-related proteins have key influence on pathogenesis and could increase the drug target space for anticancer therapies
Source: NPJ Syst Biol Appl. 2017 Jan 24;3:2. doi: 10.1038/s41540-017-0003-6 (PMC5460138; doi:10.1038/s41540-017-0003-6)
Supplement: Supplementary file 6 — Supplementary Fig. 5 [file 41540_2017_3_MOESM6_ESM.pptx]

## Slide 1
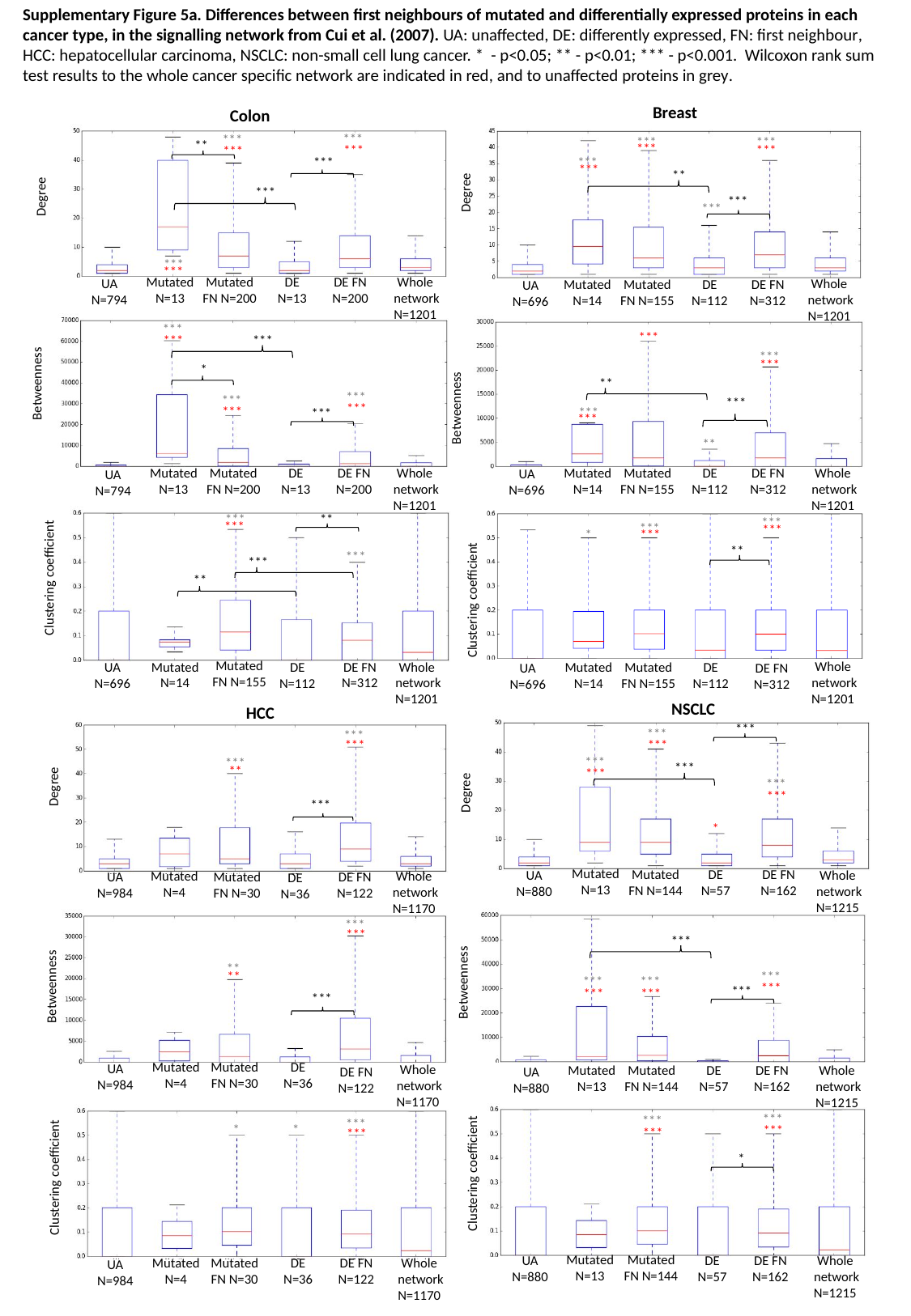

Supplementary Figure 5a. Differences between first neighbours of mutated and differentially expressed proteins in each cancer type, in the signalling network from Cui et al. (2007). UA: unaffected, DE: differently expressed, FN: first neighbour, HCC: hepatocellular carcinoma, NSCLC: non-small cell lung cancer. * - p<0.05; ** - p<0.01; *** - p<0.001. Wilcoxon rank sum test results to the whole cancer specific network are indicated in red, and to unaffected proteins in grey.
Breast
Colon
***
***
***
***
**
***
***
***
***
***
***
***
**
***
Degree
Degree
***
***
***
***
DE FN
N=200
Whole
 network
N=1201
Mutated
N=13
Mutated
FN N=200
DE
N=13
Whole
 network
N=1201
UA
N=794
DE FN
N=312
Mutated
N=14
Mutated
FN N=155
DE
N=112
UA
N=696
***
***
***
***
***
***
*
**
Betweenness
***
***
***
Betweenness
***
***
***
***
***
**
DE FN
N=200
DE FN
N=312
Whole
 network
N=1201
Mutated
N=13
Mutated
FN N=200
Whole
 network
N=1201
Mutated
N=14
Mutated
FN N=155
DE
N=13
DE
N=112
UA
N=696
UA
N=794
**
***
***
***
***
***
*
***
**
***
***
Clustering coefficient
**
Clustering coefficient
Mutated
FN N=155
Whole
 network
N=1201
DE FN
N=312
Whole
 network
N=1201
Mutated
N=14
DE
N=112
Mutated
N=14
Mutated
FN N=155
DE
N=112
UA
N=696
UA
N=696
DE FN
N=312
NSCLC
HCC
***
***
***
***
***
***
***
***
**
***
***
Degree
Degree
***
***
*
Mutated
N=13
DE FN
N=162
Mutated
FN N=144
DE
N=57
Whole
 network
N=1215
UA
N=880
Mutated
N=4
Whole
 network
N=1170
Mutated
FN N=30
UA
N=984
DE FN
N=122
DE
N=36
***
***
***
**
***
**
***
***
Betweenness
Betweenness
***
***
***
***
***
Mutated
N=4
Mutated
FN N=30
DE
N=36
UA
N=984
Whole
 network
N=1170
DE FN
N=162
Whole
 network
N=1215
Mutated
N=13
Mutated
FN N=144
DE
N=57
UA
N=880
DE FN
N=122
***
***
***
*
*
***
***
***
*
Clustering coefficient
Clustering coefficient
Mutated
N=13
Mutated
FN N=144
UA
N=880
DE FN
N=162
Whole
 network
N=1215
DE
N=57
Mutated
N=4
Mutated
FN N=30
DE FN
N=122
Whole
 network
N=1170
DE
N=36
UA
N=984

## Slide 2
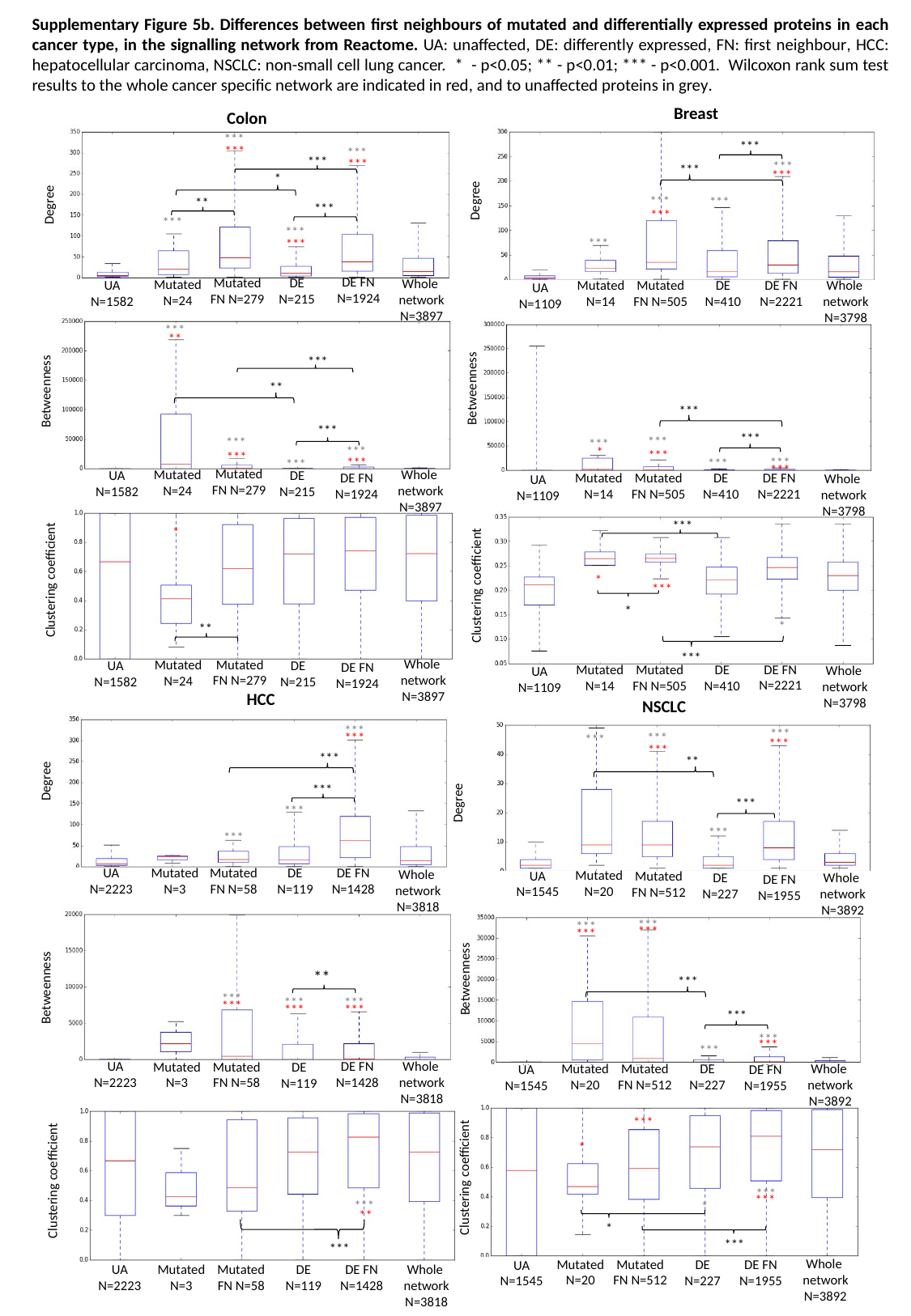

Supplementary Figure 5b. Differences between first neighbours of mutated and differentially expressed proteins in each cancer type, in the signalling network from Reactome. UA: unaffected, DE: differently expressed, FN: first neighbour, HCC: hepatocellular carcinoma, NSCLC: non-small cell lung cancer. * - p<0.05; ** - p<0.01; *** - p<0.001. Wilcoxon rank sum test results to the whole cancer specific network are indicated in red, and to unaffected proteins in grey.
Breast
Colon
***
***
***
***
***
***
***
***
***
*
Degree
***
***
**
Degree
***
***
***
***
***
***
DE FN
N=1924
DE
N=215
Mutated
FN N=279
Whole
network
N=3897
Mutated
N=24
UA
N=1582
DE FN
N=2221
Whole
network
N=3798
Mutated
N=14
Mutated
FN N=505
DE
N=410
UA
N=1109
***
**
***
**
Betweenness
Betweenness
***
***
***
***
***
***
***
*
***
***
***
***
***
***
***
Mutated
FN N=279
Mutated
N=24
Whole
network
N=3897
UA
N=1582
DE
N=215
DE FN
N=1924
DE FN
N=2221
Mutated
N=14
Mutated
FN N=505
DE
N=410
Whole
network
N=3798
UA
N=1109
***
*
*
Clustering coefficient
Clustering coefficient
***
*
*
**
***
Whole
network
N=3897
Mutated
FN N=279
Mutated
N=24
DE
N=215
UA
N=1582
DE FN
N=1924
DE FN
N=2221
Mutated
N=14
Mutated
FN N=505
DE
N=410
Whole
network
N=3798
UA
N=1109
HCC
NSCLC
***
***
***
***
***
***
***
***
**
Degree
***
Degree
***
***
***
***
DE FN
N=1428
UA
N=2223
Mutated
N=3
DE
N=119
Mutated
FN N=58
Whole
network
N=3818
Mutated
N=20
UA
N=1545
Mutated
FN N=512
DE
N=227
Whole
network
N=3892
DE FN
N=1955
***
***
***
***
**
Betweenness
***
Betweenness
***
***
***
***
***
***
***
***
***
***
UA
N=2223
DE FN
N=1428
Whole
network
N=3818
Mutated
N=3
Mutated
FN N=58
DE
N=119
Whole
network
N=3892
Mutated
N=20
Mutated
FN N=512
DE
N=227
DE FN
N=1955
UA
N=1545
***
*
Clustering coefficient
Clustering coefficient
***
***
***
*
**
*
***
***
Whole
network
N=3892
Mutated
N=20
Mutated
FN N=512
DE
N=227
DE FN
N=1955
UA
N=1545
UA
N=2223
DE FN
N=1428
Whole
network
N=3818
Mutated
N=3
Mutated
FN N=58
DE
N=119

## Slide 3
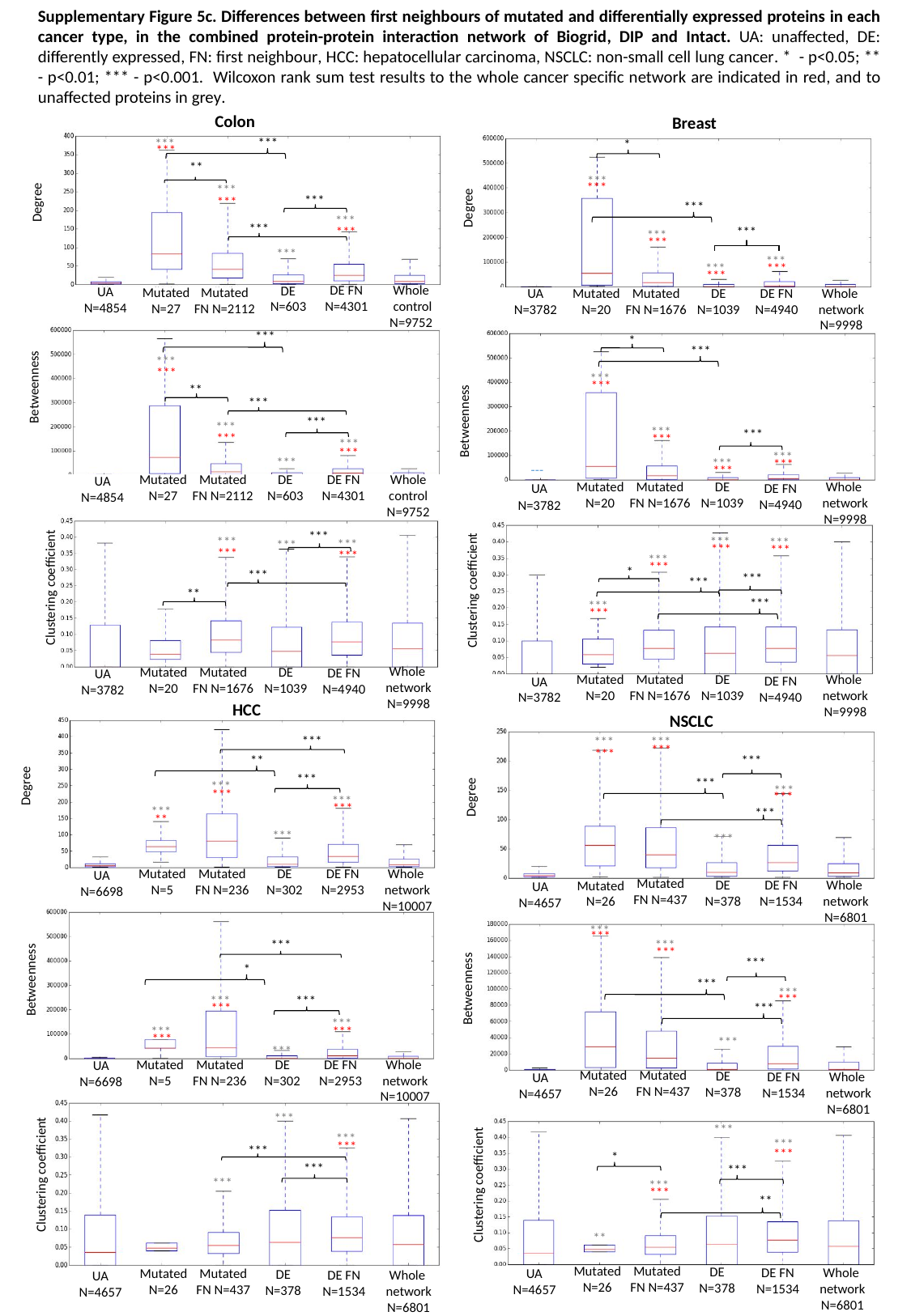

Supplementary Figure 5c. Differences between first neighbours of mutated and differentially expressed proteins in each cancer type, in the combined protein-protein interaction network of Biogrid, DIP and Intact. UA: unaffected, DE: differently expressed, FN: first neighbour, HCC: hepatocellular carcinoma, NSCLC: non-small cell lung cancer. * - p<0.05; ** - p<0.01; *** - p<0.001. Wilcoxon rank sum test results to the whole cancer specific network are indicated in red, and to unaffected proteins in grey.
Colon
Breast
***
***
*
***
**
***
***
***
***
***
Degree
***
Degree
***
***
***
***
***
***
***
***
***
***
***
DE FN
N=4301
Whole
 control
N=9752
DE
N=603
UA
N=4854
Mutated
FN N=2112
Mutated
N=27
DE FN
N=4940
Whole
network
N=9998
Mutated
N=20
Mutated
FN N=1676
DE
N=1039
UA
N=3782
***
*
***
***
***
***
***
Betweenness
**
***
Betweenness
***
***
***
***
***
***
***
***
***
***
***
***
---
***
DE FN
N=4301
Whole
control
N=9752
Mutated
N=27
Mutated
FN N=2112
DE
N=603
UA
N=4854
Whole
network
N=9998
Mutated
N=20
Mutated
FN N=1676
DE
N=1039
DE FN
N=4940
UA
N=3782
***
***
***
***
***
***
***
***
***
***
***
***
*
***
***
***
Clustering coefficient
Clustering coefficient
**
***
***
***
Whole
network
N=9998
Mutated
N=20
Mutated
FN N=1676
DE
N=1039
DE FN
N=4940
UA
N=3782
Whole
network
N=9998
Mutated
N=20
Mutated
FN N=1676
DE
N=1039
DE FN
N=4940
UA
N=3782
HCC
NSCLC
***
***
***
***
***
***
**
***
***
Degree
***
***
***
***
Degree
***
***
***
***
**
***
***
DE FN
N=2953
Whole
network
N=10007
Mutated
N=5
Mutated
FN N=236
DE
N=302
UA
N=6698
Mutated
FN N=437
DE FN
N=1534
Whole
network
N=6801
DE
N=378
Mutated
N=26
UA
N=4657
***
***
***
***
***
***
*
Betweenness
***
Betweenness
***
***
***
***
***
***
***
***
***
***
***
***
DE FN
N=2953
Whole
network
N=10007
Mutated
N=5
Mutated
FN N=236
DE
N=302
UA
N=6698
Mutated
N=26
Mutated
FN N=437
DE
N=378
DE FN
N=1534
Whole
network
N=6801
UA
N=4657
***
***
***
***
***
***
***
*
***
***
Clustering coefficient
***
***
Clustering coefficient
***
**
**
Mutated
N=26
Mutated
FN N=437
DE
N=378
DE FN
N=1534
Whole
network
N=6801
Mutated
N=26
Mutated
FN N=437
UA
N=4657
DE
N=378
DE FN
N=1534
Whole
network
N=6801
UA
N=4657

## Slide 4
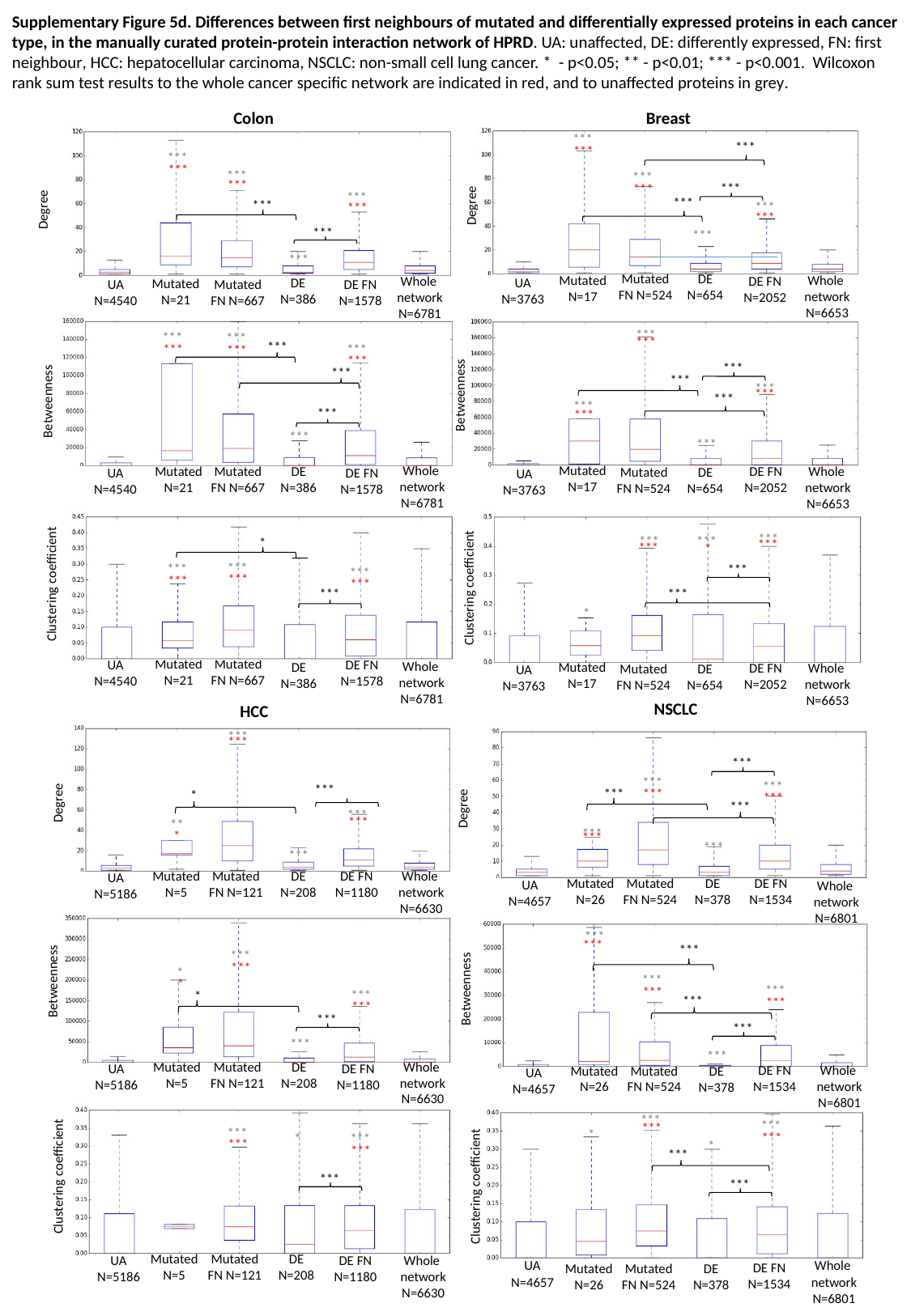

DE
N=654
Supplementary Figure 5d. Differences between first neighbours of mutated and differentially expressed proteins in each cancer type, in the manually curated protein-protein interaction network of HPRD. UA: unaffected, DE: differently expressed, FN: first neighbour, HCC: hepatocellular carcinoma, NSCLC: non-small cell lung cancer. * - p<0.05; ** - p<0.01; *** - p<0.001. Wilcoxon rank sum test results to the whole cancer specific network are indicated in red, and to unaffected proteins in grey.
Colon
Breast
***
***
***
***
***
***
***
***
***
***
***
***
***
Degree
***
***
Degree
***
***
***
***
DE
N=654
Mutated
FN N=524
Whole
network
N=6653
Mutated
N=17
Whole
network
N=6781
DE FN
N=2052
DE
N=386
UA
N=3763
Mutated
N=21
DE FN
N=1578
UA
N=4540
Mutated
FN N=667
***
***
***
***
***
***
***
***
***
***
***
***
***
***
Betweenness
***
Betweenness
***
***
***
***
***
Mutated
N=17
Whole
network
N=6781
DE
N=386
Mutated
N=21
Mutated
FN N=667
Whole
network
N=6653
DE FN
N=2052
Mutated
FN N=524
DE
N=654
UA
N=4540
DE FN
N=1578
UA
N=3763
***
***
***
*
***
***
*
***
***
***
***
***
***
***
Clustering coefficient
Clustering coefficient
***
***
*
Mutated
N=21
Mutated
FN N=667
UA
N=4540
DE FN
N=1578
Whole
network
N=6781
Mutated
N=17
DE
N=386
Whole
network
N=6653
DE FN
N=2052
Mutated
FN N=524
DE
N=654
UA
N=3763
NSCLC
HCC
***
***
***
***
***
***
***
***
*
***
Degree
***
Degree
***
***
**
***
*
***
***
***
DE FN
N=1180
Whole
network
N=6630
Mutated
N=5
Mutated
FN N=121
DE
N=208
UA
N=5186
DE FN
N=1534
Mutated
N=26
Mutated
FN N=524
DE
N=378
UA
N=4657
Whole
network
N=6801
***
***
***
***
***
*
***
Betweenness
*
Betweenness
***
***
***
*
***
***
***
***
***
***
***
Whole
network
N=6630
Mutated
N=5
Mutated
FN N=121
DE
N=208
DE FN
N=1180
UA
N=5186
DE FN
N=1534
Whole
network
N=6801
Mutated
N=26
Mutated
FN N=524
DE
N=378
UA
N=4657
***
***
***
***
*
***
*
***
***
*
***
***
Clustering coefficient
***
***
Clustering coefficient
Mutated
N=5
Mutated
FN N=121
DE
N=208
UA
N=5186
DE FN
N=1180
Whole
network
N=6630
UA
N=4657
Whole
network
N=6801
DE FN
N=1534
Mutated
N=26
Mutated
FN N=524
DE
N=378
