# Supplementary material for: Neighbours of cancer-related proteins have key influence on pathogenesis and could increase the drug target space for anticancer therapies
Source: NPJ Syst Biol Appl. 2017 Jan 24;3:2. doi: 10.1038/s41540-017-0003-6 (PMC5460138; doi:10.1038/s41540-017-0003-6)
Supplement: Supplementary file 7 — Supplementary Fig. 6 [file 41540_2017_3_MOESM7_ESM.pptx]

## Slide 1
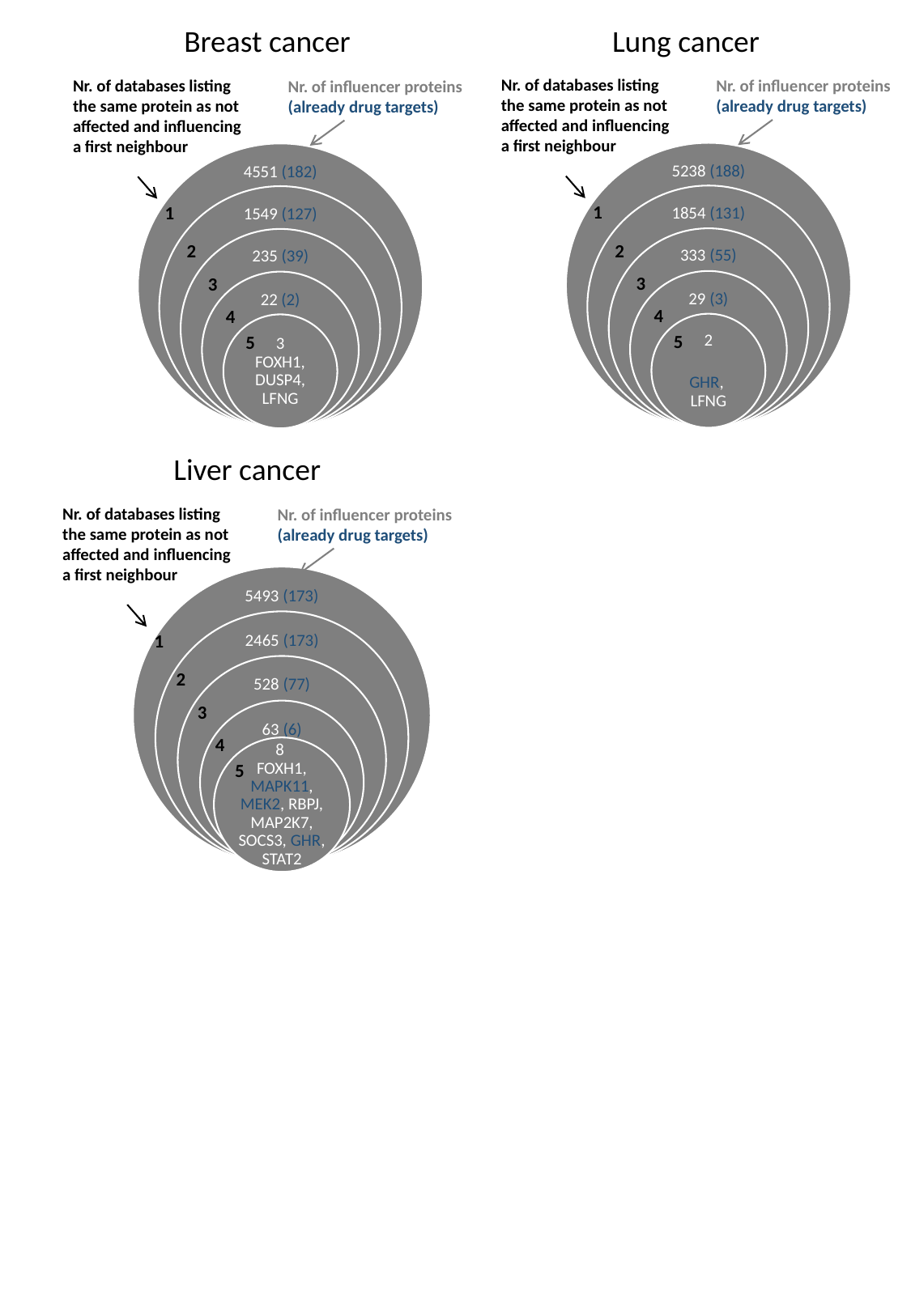

Lung cancer
Breast cancer
Nr. of databases listing the same protein as not affected and influencing a first neighbour
Nr. of databases listing the same protein as not affected and influencing a first neighbour
Nr. of influencer proteins (already drug targets)
Nr. of influencer proteins (already drug targets)
1
1
2
2
3
3
4
4
5
5
Liver cancer
Nr. of databases listing the same protein as not affected and influencing a first neighbour
Nr. of influencer proteins (already drug targets)
1
2
3
4
5
